# Tracing anti-cancer and cancer-promoting actions of all-trans retinoic acid in breast cancer to a RARa epigenetic mechanism of mammary epithelial cell fate

**DOI:** 10.18632/oncotarget.13500

**Published:** 2016-11-22

**Authors:** Stefano Rossetti, MingQiang Ren, Nicolo Visconti, Francesca Corlazzoli, Vincenzo Gagliostro, Giulia Somenzi, Jin Yao, Yijun Sun, Nicoletta Sacchi

**Affiliations:** ^1^ Department of Cancer Genetics, Roswell Park Cancer Institute, Buffalo, NY 14263, USA; ^2^ The State University of New York at Buffalo, Center of Excellence in Bioinformatics and Life Sciences, Buffalo, NY 14203, USA

**Keywords:** retinoic acid (RA), RARA, epigenetic transcriptional regulation, mammary epithelial cell fate decisions, breast cancer

## Abstract

A hallmark of cancer cells is the ability to evade the growth inhibitory/pro-apoptotic action of physiological all-*trans* retinoic acid (RA) signal, the bioactive derivative of Vitamin A. However, as we and others reported, RA can also promote cancer cell growth and invasion. Here we show that anticancer and cancer-promoting RA actions in breast cancer have roots in a mechanism of mammary epithelial cell morphogenesis that involves both transcriptional (epigenetic) and non-transcriptional RARα (RARA) functions. We found that the mammary epithelial cell-context specific degree of functionality of the RARA transcriptional (epigenetic) component of this mechanism, by tuning the effects of the non-transcriptional RARA component, determines different cell fate decisions during mammary morphogenesis. Indeed, factors that hamper the RARA epigenetic function make physiological RA drive aberrant morphogenesis via non-transcriptional RARA, thus leading to cell transformation. Remarkably, also the cell context-specific degree of functionality of the RARA epigenetic component retained by breast cancer cells is critical to determine cell fate decisions in response to physiological as well as supraphysiological RA variation. Overall this study supports the proof of principle that the epigenetic functional plasticity of the mammary epithelial cell RARA mechanism, which is essential for normal morphogenetic processes, is necessary to deter breast cancer onset/progression consequent to the insidious action of physiological RA.

## INTRODUCTION

Epigenetic regulation of transcription plays a significant role in development by regulating major developmental processes, such as X-inactivation, imprinting, and spatiotemporal activation of homeobox genes [1]. In the course of animal evolution, development became dependent also on an environmental factor from plant and animal sources, all-*trans* retinoic acid (hereafter RA), that epigenetically regulates transcription by binding nuclear RA receptors (RARs) [2–5]. In response to RA variation RARs, as heterodimers with rexinoid receptors (RXRs) [6], by recruiting chromatin coactivator or corepressor regulatory complexes and chromatin modifying enzymes, finely regulate the chromatin at genes characterized mostly, but not exclusively, by specific RA responsive elements (RAREs) [7, 8], thus creating a connection between this environmental signal and the genome [9, 10].

Fine-tuning the balance between active and repressed chromatin is one of the most crucial tasks of cell fate decision during development. Genome-wide transcriptional regulation in response to precise spatiotemporal variation of physiological RA – which, as a morphogen, determines cell fate in a concentration-dependent manner – has been considered an essential underlying molecular mechanism impacting several facets of development: body plan, organogenesis, morphogenesis, differentiation and tissue homeostasis [2, 4, 11–13]. Indeed too much or too little RA dramatically hinders developmental processes and produces teratogenic effects [14]. Since generation of precise RA level variation is of essence for determining cell fate decisions during normal development, animal cells evolved mechanisms to regulate transcriptionally also genes controlling the metabolism of RA and its precursors, including Retinol/Vitamin A [15]. Interestingly, animal evolutionary studies identified molecular vestiges of a two-module RA mechanism encompassing a ‘RA metabolic module’ integrated with a ‘RA signaling module’ regulating gene expression [16].

In specific developmental contexts, the RA-RAR mechanism is connected with different upstream and downstream nuclear receptors. For example, in epithelial cells of the mammary gland, nuclear RARα (RARA), on one hand, is directly transcriptionally regulated via estrogen receptor α (ERA) [17] and, on the other hand, directly regulates the transcription of downstream RARs, including the tumor suppressor RARβ2 (RARB2) [18], thus establishing developmental-specific transcriptional cascades epigenetically regulated by hormone and RA signals. Moreover, RA controls other transcriptional signaling pathways via different nuclear receptors, such as peroxisome proliferator-activated receptor β/δ (PPARD) [19, 20] and chicken ovalbumin upstream promoter transcription factor 2 (COUP-TFII) [21]. There is compelling evidence that RA can also regulate in a non-transcriptional fashion different kinases either by direct interaction, as in the case of protein kinase C alpha (PKCA) [22, 23], or via RARA, as in the case of phosphatidyl inositide 3 kinase (PI3K) [24], thus establishing a cross-talk between different RA signaling pathways [25, 26].

This complexity, which possibly evolved to suit specific developmental and physiological needs during animal evolution, emerges also in cancer. Normal cells, when turn malignant, grow and invade at distant sites unchecked by growth-inhibitory and pro-apoptotic physiological signals [27], including physiological RA signal. There is *in vivo* mechanistic evidence that preventing physiological RA from activating wild type RARA transcriptional function in the mammary gland induces typical breast cancer features, such as aberrant ductal morphology and excessive cell proliferation [28]. Similarly, *in vitro* studies, including ours, indicate that functional inhibition of wild type RARA transcriptional activity in mammary epithelial cells changes physiological RA action from morphogenetic to cancer-promoting [18, 29–33]. Consistently, breast cancer cells without RARA mutations, but with epigenetic signs of functional inhibition of RARA transcriptional activity, form tumors under *in vivo* physiological RA conditions [34, 35]. As reported in clinical trials for other cancers [36], we found that supraphysiological RA exerts paradoxical opposing actions also on breast cancer cell growth, depending on the level/functionality of wild type RARA among different breast cancer cell contexts, as well as within the same breast cancer cell context [34, 35, 37].

In this study, we traced both anti-cancer and cancer-promoting actions of physiological and supraphysiological RA in breast cancer cells to the plasticity of a RARA epigenetic mechanism of normal mammary epithelial cell morphogenesis. Different from the two-module mechanism inferred from evolutionary studies [16], the mechanism that we show here encompasses a ‘RA metabolic module’ integrated not only with the ‘RARA transcriptional module’, which exerts a genome-wide epigenetic control of RARA-target genes, but also with a ‘non-transcriptional RARA module’, which controls PI3K kinase signaling pathways. All three modules are indispensable for normal morphogenesis of lumen-enclosing epithelial monolayers typical of the mammary gland tree. However, the plasticity of the RARA transcriptional arm, which is the epigenetic regulatory component of this mechanism, is critical to determine the different actions of physiological RA and consequent cell fate decisions during normal morphogenesis. Conversely, as shown here in a mammary epithelial cell context, a dysfunctional RARA transcriptional arm can determine: a) the physiological RA cancer-promoting action leading to aberrant mammary morphogenesis, which is a feature of breast cancer initiation, b) differential cancer-promoting actions of physiological (endogenous) RA during cancer progression, and even c) paradoxical anticancer and cancer-promoting actions of supraphysiological (exogenous) RA used for cancer treatment. Overall, this study supports the proof of concept that RA breast cancer-promoting action has roots in a cell-autonomous RARA epigenetic mechanism of mammary morphogenesis.

## RESULTS

### Evidence that regulation of breast cancer cell growth by RA implicates, in addition to the RARA transcriptional function, another RARA function

RA, which is considered a powerful anti-cancer agent, can paradoxically promote cancer growth and invasion [36, 38, 39]. Our previous studies, aimed at unraveling mechanism(s) of the different RA actions in breast morphogenesis and tumorigenesis, pointed to the involvement of both an epigenetic component and different RA signaling pathways [18, 29, 34, 35, 37, 40]. The *in vitro* and *in vivo* studies reported hereafter provide the first clues of a cell-autonomous epigenetic mechanism that can explain both anticancer and cancer-promoting RA actions.

**Figure 1:**
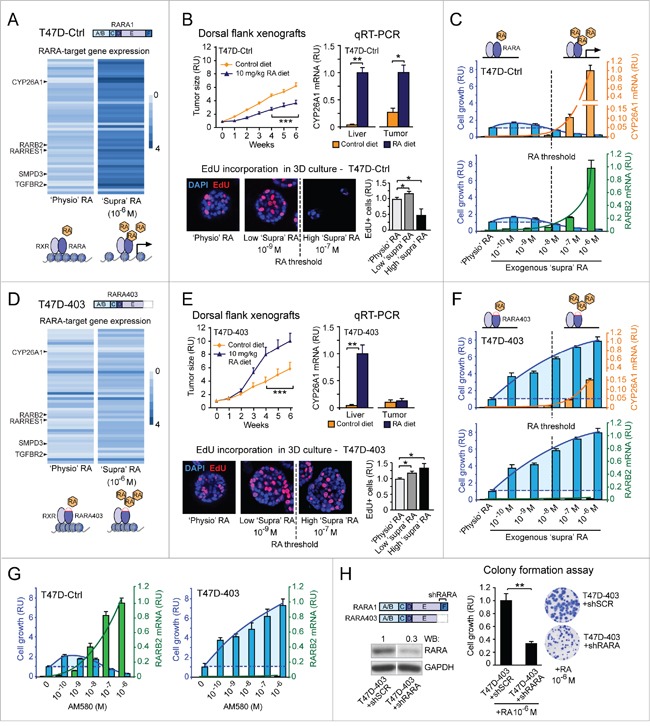
Evidence that regulation of breast cancer cell growth by RA implicates, in addition to the RARA transcriptional function, another RARA function **A**. Analysis of human gene expression microarrays shows that the transcription of RARA-target genes is repressed under ‘physiological’ (‘physio’) RA conditions, but can be reactivated by ‘supraphysiological’ (‘supra’) RA (10^-6^ M) in T47D^Ctrl^ cells. **B**. T47D^Ctrl^ xenograft tumors are growth-inhibited in nude mice fed a RA-enriched diet relative to a control diet (***p<0.001) (top, left), consistent with CYP26A1 transcript induction in both mice liver and tumors (*p<0.05, **p<0.01) (top, right). 3D T47D^Ctrl^ growth (assessed by EdU incorporation) is promoted by low ‘supraphysiological’ RA (10^-9^ M) (*p<0.05), but inhibited by high RA (10^-7^ M) (*p<0.05). **C**. ‘Supraphysiological’ RA promotes 2D T47D^Ctrl^ cell growth (assessed by colony formation) at concentrations below the threshold required for induction of CYP26A1 and RARB2 transcripts (measured by qRT-PCR), but inhibits growth above this threshold (CYP26A1: RA 10^-7^M = **p<0.01, RA 10^-6^M = *p<0.05; RARB2: RA 10^-7^M = ***p<0.001, RA 10^-6^M = **p<0.01; growth: RA≤10^-9^ M = *p<0.05, RA≥10^-7^M = ***p<0.001; relative to ‘physio’ RA). **D**. In T47D^403^ cells, ‘supraphysiological’ RA does not re-activate RARA-target gene expression as it does in T47D^Ctrl^ cells. **E**. The RA-enriched diet promotes T47D^403^ xenograft tumor growth (***p<0.001) (top, left). This diet induces CYP26A1 transcript in mice liver but not in xenograft tumors (**p<0.01) (top, right). Both low and high ‘supraphysiological’ RA promote 3D T47D^403^ growth (*p<0.05) (bottom). **F**. ‘Supraphysiological’ RA induces CYP26A1 (RA 10^-7^M = *p<0.05, RA 10^-6^M = **p<0.01) and RARB2 (RA 10^-7^M = ***p<0.001, RA 10^-6^M = **p<0.01) significantly less relative to T47D^Ctrl^ cells. At all concentrations, RA promotes 2D T47D^403^ growth (***p<0.001, relative to ‘physio’ RA). **G**. The RARA agonist AM580 recapitulates the effects of RA on cell growth (T47D^Ctrl^: **p<0.01 and T47D^403^: ***p<0.01, relative to untreated) and RARB2 transcription (T47D^Ctrl^: AM580≥10^-8^M = **p<0.01, relative to untreated; T47D^403^: **p<0.01, relative to T47D^Ctrl^). **H**. Stable knock down of wild type RARA in T47D^403^ cells (left) counteracts RA-induced cell growth (assessed by colony formation assay, right) (**p<0.01). Student's t-test was used for *in vitro* studies and ANOVA for *in vivo* studies. RU= relative units.

By global gene expression microarray analysis we found that in breast cancer cells (T47D^Ctrl^) grown under ‘physiological’ (‘physio’) RA culture conditions, many RARA-target genes are in a repressed transcriptional state marked by epigenetic histone modifications, but are transcriptionally responsive to high ‘supraphysiological’ (‘supra’) exogenous RA (10^-6^M) in the culture medium (Figure 1A, based on Supplementary Table S1, and Supplementary Figure S1). In nude mice fed a normal control diet, T47D^Ctrl^ cells grow as xenograft tumors, indicative of acquisition of resistance to physiological RA anticancer action, but are growth-inhibited in mice fed a RA-enriched diet (Figure 1B, top left). The transcription of cytochrome P450 26A1 (CYP26A1), a prototypic RARA-target gene used as an indicator of RA level variation [41], is induced by a RA-enriched diet in both liver and T47D^Ctrl^ tumors (Figure 1B, top right). To rapidly assess the effects of increasing ‘supraphysiological’ RA (from 10^-9^M to 10^-7^M) on T47D^Ctrl^ cell growth, we grew cells in three-dimensional (3D) culture (Matrigel), where they form amorphous structures typical of breast tumorigenic cells [42]. Both in 3D (Figure 1B, bottom) and two-dimensional (2D) (Figure 1C) culture, T47D^Ctrl^ cell growth is promoted by RA only below the 10^-8^ M threshold level, where there is no, or insufficient, transcriptional activation of both CYP26A1 and the tumor suppressor RARB2 gene (Figure 1C).

Ectopic expression of the dominant negative RARA403 mutant in the T47D context (T47D^403^) further represses the already repressed transcriptional state of RARA-targets (Figure 1D, based on Supplementary Table S1, and Supplementary Figure S1). This explains why CYP26A1 remains transcriptionally repressed in T47D^403^ tumors, but not in the liver of mice fed the RA-enriched diet (Figure 1E, top right), even when tumors grow significantly more than in mice fed a normal diet (Figure 1E, top left). Likewise, ‘supraphysiological’ RA, both below and above the 10^-8^ M RA threshold, promotes both 3D (Figure 1E, bottom) and 2D (Figure 1F) growth of T47D^403^ cells, because it cannot reactivate effectively the transcription of RARA-target genes (Figure 1F). Indeed, even at high ‘supraphysiological’ RA (>10^-8^M), both CYP26A1 and RARB2 transcripts are induced significantly less in T47D^403^ than in T47D^Ctrl^ (Figure 1F).

We excluded that the RA growth-promoting action is limited to either the specific RARA403 mutation or the T74D breast cancer cell context, because we observed RA induction of cell growth also when we took into consideration another dominant negative RARA allele (RARAG303E) (Figure S2A) and another breast cancer cell context (MCF7) (Supplementary Figure S3).

**Figure 2:**
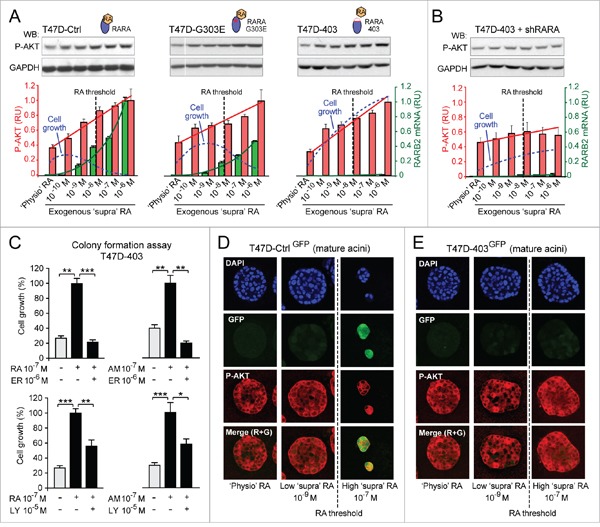
Evidence of combinatorial effects of both RA-RARA-mediated transcriptional activation and RA-RARA-mediated PI3K activation on breast cancer cell growth **A**. In the T47D context, depending on the degree of functionality of RARA transcriptional activity (T47D^Ctrl^ > T47D^G303E^ > T47D^403^), the differential growth (blue dotted line) induced by increasing exogenous RA reflects the combined effects of RARA transcriptional activation (assessed by RARB2 qRT-PCR, green columns) and RARA-PI3K activation (assessed by P-AKT quantitative Western Blot, red columns). In all clones RA concentration significantly correlates with P-AKT level (r=0.945, 0.91-0.97, 95% CI) and RARB2 expression (r=0.62, 0.45-0.75, 95% CI). **B**. Stable knock down of wild type RARA in T47D^403^ cells counteracts both PI3K activation and growth promotion induced by increasing exogenous RA. **C**. Treatment with either the RARA antagonist ER50891 (ER) (top) or the PI3K inhibitor LY294002 (LY) (bottom) counteracts T47D^403^ growth induced by both RA and the RARA agonist AM580 (AM) (*p<0.05, **p<0.01, ***p<0.001). **D**. ‘Supraphysiological’ RA inhibits T47D^Ctrl^ 3D growth only at concentrations above the threshold that can induce, in addition to RARA-PI3K signaling (assessed by P-AKT), also RARA transcriptional signaling (assessed by RARE-GFP). **E**. In contrast, ‘supraphysiological’ RA, at all concentrations, only promotes T47D^403^ 3D growth, because it sustains RARA-PI3K signaling, but does not activate RARA transcriptional signaling. Significance calculated by standard linear regression (A) or Student's t-test (C).

We did also exclude that the mediator of ‘supraphysiological’ RA cancer-promoting action in breast cancer cells with inhibition of RARA transcriptional function is PPARD. As shown here by both genetic and pharmacological approaches, we discounted a role of PPARD in the T47D breast cancer cell context with maximal inhibition of RARA transcriptional function (Supplementary Figure S4A-D). Interestingly, in the course of these experiments, we instead found that RARA-specific agonists (shown here AM580) recapitulate the cancer-promoting and transcriptional effects of RA (Supplementary Figure S4E), suggesting that, in addition to RARA transcriptional function, another RARA function is involved in the control of cancer cell growth. Indeed, in T47D contexts with either wild type RARA (T47D^Ctrl^) or dominant negative mutant RARA alleles (T47D^G303E^ and T47D^403^), the dose-dependent effects of AM580 variation (Figure 1G and Supplementary Figure S2B) mirrors the dose-dependent effects of RA variation on both cell growth and RARB2 transcription (Figure 1C, bottom, Figure 1F, bottom, and Supplementary Figure S2A, bottom). Moreover, wild type RARA knock down in the T47D^403^ context with a RARA-targeting short hairpin RNA (shRNA) (Figure 1H, left) significantly counteracts cancer cell growth induced by high ‘supraphysiological’ RA (10^-6^ M) (Figure 1H, right).

These findings prompted us to search for further evidence of the “other” putative RARA function involved in the regulation of T47D breast cancer cell growth by RA.

### Evidence of combinatorial effects of both RA-RARA-mediated transcriptional activation and RA-RARA-mediated PI3K activation on breast cancer cell growth

According to literature, in cancer cells RA can activate PI3K kinase by enhancing RARA physical interaction with the PI3K catalytic subunit (p110) [24]. After identifying by immunoprecipitation a protein complex comprising wild type RARA, the regulatory PI3K subunit (p85α), and the catalytic PI3K mutant subunit (p110α) (Figure S5A, left) in the T47D cell context, we found by proximity ligation assay (PLA) that both low RA (10^-9^M) and the RARA agonist AM580 (10^-9^ M) did enhance RARA-p110α interaction (Figure S5A, right). Next, we found that after the first hour of a 72 hour-treatment of T47D^Ctrl^ cells with increasing exogenous RA (from 10^-10^M to 10^-6^M), the extent of activation of both RARA transcription (RARB2 transcript level, green) and PI3K (phosphorylation of the PI3K effector AKT, red) correlates with the growth outcome (assessed by colony formation after the 72 hour RA treatment, blue) (Figure 2A, left). As shown hereafter, the relationship between cell growth and activation of the two RARA functions in response to exogenous RA variation seems to be influenced by the cell context-specific degree of inhibition of RARA transcriptional function: mild in T47DCtrl, severe in T47DG303E, and extremely severe in T47D403 (Figure 2A). Under ‘physiological’ RA culture conditions (that is without addition of exogenous RA), T47D^Ctrl^, T47D^G303E^, and T47D^403^ cells display only a basal P-AKT level (Figure 2A). In contrast, exogenous, ‘supraphysiological’ RA variation induces a similar increase of AKT phosphorylation (P-AKT) in the three cell contexts, but differentially induces RARB2 transcript level (Figure 2A). Regression analysis shows that, in response to increasing exogenous RA, the growth outcome of T47D^Ctrl^, T47D^G303E^, and T47D^403^ reflects the combined effect of cell context-specific activation of both transcriptional and non-transcriptional RARA functions (Figure 2A). This interpretation is supported by evidence of a significantly decreased RA-induced T47D^403^ cell growth – in correlation with a reduced P-AKT level – after stable wild type RARA knock down in T47D^403^ (Figure 2B). Consistently, treatment with either the RARA antagonist ER50891 (Figure 2C, top) or the PI3K inhibitor LY294002 (Figure 2C, bottom) significantly counteracts T47D^403^ growth induced by either RA or the RARA agonist AM580. It is noteworthy to mention that also Retinol/Vitamin A (ROH), a dietary RA precursor, which activates PI3K signaling (but not RARA transcriptional signaling) in T47D^403^ cells, promotes cell growth both *in vitro* and *in vivo* (Supplementary Figure S6).

By confocal microscopy of 3D mature acini formed by T47D^Ctrl^ and T47D^403^ cells stably transfected with a RARE-GFP reporter, we could infer the combined biological effects of both transcriptional (GFP, green) and non-transcriptional (P-AKT, red) RARA functions at both ‘physiological’ and increasing ‘supraphysiological’ RA culture conditions. Only P-AKT is detectable in 3D mature T47D^Ctrl-GFP^ acini developed either in the absence or presence of low exogenous RA (10^-9^M) (Figure 2D, left and middle columns), while both GFP and P-AKT are detectable in T47D^Ctrl-GFP^ growth-inhibited acini developed in the presence of high exogenous RA (10^-7^M) (Figure 2D, right column). Conversely, T47D^403-GFP^ cells developed into 3D acini expressing only P-AKT, even at high ‘supraphysiological’ RA culture conditions (Figure 2E).

Based on these findings, breast cancer cell fate decisions seem to depend on how the biological effects of RARA-mediated transcriptional regulation of direct target genes keep in check the cancer-promoting effects of RARA-mediated activation of PI3K effectors in response to RA variation. Thus, we set out to substantiate our hypothesis with additional experiments shown hereafter.

### RA promotes breast cancer cell invasion via RARA-PI3K when it cannot reactivate transcriptionally silent tumor suppressor RARA-target genes: A proof of concept

**Figure 3:**
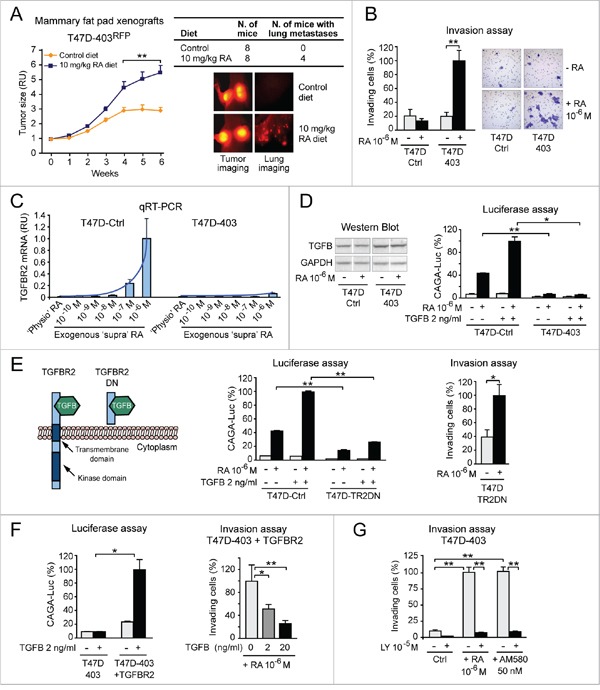
RA promotes breast cancer cell invasion via RARA-PI3K when it cannot reactivate transcriptionally silent tumor suppressor RARA-target genes: A proof of concept **A**. A RA-enriched diet promotes not only the growth of RFP-positive T47D^403^ xenograft tumors (left), but also the formation of lung metastases (right) (**p<0.01). **B**. High ‘supraphysiological’ RA promotes T47D^403^ invasion in the Boyden chamber assay (Giemsa-stained invading cells in representative fields are shown on the right) (**p<0.01). **C**. ‘Supraphysiological’ RA induces TGFBR2 transcript significantly less in T47D^403^ than in T47D^Ctrl^ (RA≥10^-7^M = *p<0.05) **D**. Consistently, T47D^403^ cells cannot transduce the signal of both endogenous TGFB (detected by western blot in D, left) and exogenous TGFB, alone or in combination with exogenous RA (shown by CAGA-luc assay in D, right) (*p<0.05, **p<0.01). **E**. Stable expression of a TGFBR2 dominant negative (DN) mutant (left) in T47D^Ctrl^ cells (T47D^TR2DN^), by inhibiting TGFB signaling (assessed by CAGA-luc assay, middle), makes cells invade more in response to exogenous RA (right) (*p<0.05, **p<0.01). **F**. Conversely, stable expression of TGFBR2 in T47D^403^ cells, by re-enabling TGFB signaling (CAGA-luc assay, left), counteracts RA-induced cell invasion (right) (*p<0.05, **p<0.01). **G**. RA-induced T47D^403^ cell invasion implicates activation of RARA-PI3K signaling, because PI3K inhibition by LY294002 (LY) significantly counteracts cell invasion induced by either exogenous RA or the RARA agonist AM580 (**p<0.01). Student's t-test was used for *in vitro* studies and ANOVA for *in vivo* studies.

*In vivo* experiments show that a RA-enriched diet promotes not only tumor growth (Figure 3A, left), but also invasion of T47D^403^ cells expressing red fluorescent protein (RFP) (Figure 3A, right). *In vitro*, high ‘supraphysiological’ RA (10^-6^ M) significantly promotes T47D^403^ invasion (Figure 3B) in correlation with epigenetic transcriptional repression of tumor suppressor RARA-targets, including the transforming growth factor β (TGFB) receptor 2 (TGFBR2) (Figure 1D and Supplementary Figure S1). Indeed, TGFBR2 transcription remains epigenetically repressed both in the T47D^Ctrl^ and T47D^403^ cell contexts at no/low exogenous RA, but it is still amenable of being reactivated by high exogenous RA (>10^-8^M) in T47D^Ctrl^ cells (Figure 3C), confirming that the epigenetic TGFBR2 transcriptional repression in the T47D^403^ cell context is more severe than in T47D^Ctrl^ (Supplementary Figure S1). In response to high RA, T47D^Ctrl^ cells – but not T47D^403^ cells – transduce the signal of both endogenous TGFB (detected by western blot, Figure 3D, left) and exogenous TGFB (2 ng/ml) (Figure 3D, right).

To mechanistically test whether the activation of TGFB-TGFBR2 signaling pathway contributes to deter RA pro-invasive action, we functionally inhibited TGFBR2 in the T47D^Ctrl^ cell context by stably expressing a TGFBR2 dominant negative mutant (TR2DN) that cannot transduce TGFB signal (scheme in Figure 3E, left) [43]. High RA (10^-6^M), by inducing TGFB signaling significantly less in T47D^TR2DN^ than in T47D^Ctrl^ (Figure 3E, middle), promotes T47D^TR2DN^ cell invasion (Figure 3E, right). Conversely, ectopic expression of TGFBR2 in the T47D^403^ cell context, by re-enabling TGFB signaling pathway (Figure 3F, left), counteracts RA-induced cell invasion (Figure 3F, right). Finally, we found that the PI3K inhibitor LY294002 counteracts T47D^403^ cell invasion induced by either RA (10^-6^M), or the RARA-specific agonist AM580 (50 nM) (Figure 3G).

These findings support the proof of concept that RA exerts a breast cancer-promoting action whenever, due to epigenetic repression, RARA-transcriptionally-regulated tumor suppressor signaling pathways (e.g. TGFB-TGFBR2) fail to counteract the effects of RARA-PI3K/AKT signaling pathway.

### Both cell context-specific physiological endogenous RA synthesis and transcriptional functionality of RARA differentially determine breast cancer cell fate

As shown before, the breast cancer cell context-specific RARA transcriptional functionality seems to be critical for determining cell fate decisions in response to ‘supraphysiological’ RA variation. By the same token, due to heterogeneous expression of aldehyde dehydrogenase (ALDH), the enzyme involved in physiological RA synthesis [44], within a breast cancer cell population [45], we set out to test if breast cancer cell fate decisions may depend on both the cell context-specific transcriptional functionality of RARA and physiological endogenous RA synthesis variation.

**Figure 4:**
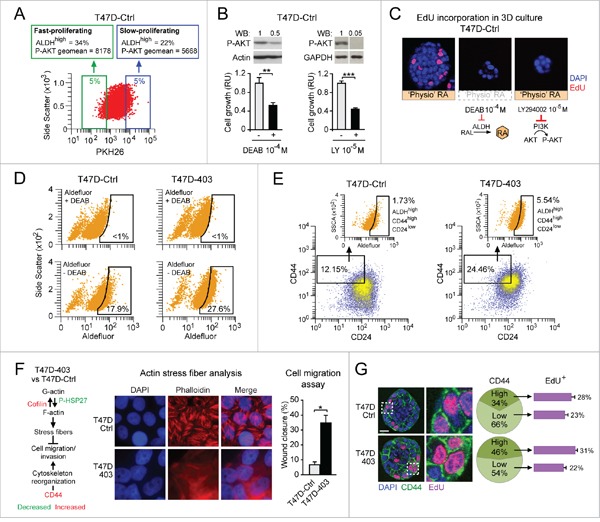
Both cell context-specific physiological endogenous RA synthesis and transcriptional functionality of RARA differentially determine breast cancer cell fate **A**. Cytofluorimetric analysis of PKH26-labeled T47D^Ctrl^ cells shows that the fast-proliferating cell subset (green frame) contains more cells with high ALDH activity (ALDH^high^ cells, detected by Aldefluor staining) and expresses more P-AKT relative to the slow-proliferating cell subset (blue frame). **B**. Inhibition of either ALDH-mediated RA synthesis with DEAB (left), or PI3K activity with LY294002 (right) results in a decrease of both P-AKT level (top) and cell proliferation (bottom) (**p<0.01, ***p<0.001). **C**. Treatment with either DEAB or LY294002 inhibits T47D^Ctrl^ proliferation also in 3D culture. **D-E**. Increased inhibition of RARA transcriptional function in T47D^403^ cells leads to expansion of both ALDH^high^ (D) and ALDH^high^/CD44^high^/CD24^low^ (E) subpopulations relative to T47D^Ctrl^. **F**. Global gene/protein expression analyses highlight pro-invasive molecular changes in T47D^403^ vs. T47D^Ctrl^ (left). Consistently, T47D^403^ cells show defective phalloidin-stained actin stress fibers (middle) and increased cell migration in the wound healing assay (right) (*p<0.05). G. Confocal analysis shows that 3D T47D^403^ acini have a higher proportion of CD44^high^ cells relative to 3D T47D^Ctrl^ acini (**p<0.01), and that the T47D^403^ CD44^high^ cell subpopulation contains more proliferating (EdU-positive) cells than the CD44^low^ cell subpopulation (**p<0.01). Significance calculated by Student's t-test.

First, by labeling T47D^Ctrl^ cells with PKH26, a fluorescent cell linker that – by being more and more diluted after each cell division – can discriminate between slow- and fast-proliferating cells (Figure 4A, bottom), we found that the fast-proliferating cell subset (low PKH26, green frame) contains ∼50% more cells with high ALDH activity (ALDH^high^ cells, detected by Aldefluor staining) and expresses ∼45% more P-AKT (assessed by immunostaining) relative to the slow-proliferating cell subset (high PKH26, blue frame) (Figure 4A, top). Inhibition of either ALDH-mediated RA synthesis with DEAB (Figure 4B, left), or PI3K activity with LY294002 (Figure 4B, right), results in a decrease of both P-AKT level and cell proliferation. Similarly, both DEAB and LY294002 severely hamper 3D T47D^Ctrl^ acinar growth in Matrigel (Figure 4C). Thus, ALDH-mediated physiological RA synthesis contributes to promote T47D^Ctrl^ cell proliferation by activating the RARA-mediated PI3K/AKT signaling pathway.

Second, we found that the cell context-specific degree of inhibition of RARA transcriptional function affects the size of the subset of cells with higher ALDH activity because, by Aldefluor cytofluorimetric analysis, we detected more ALDH^high^ cells in T47D^403^ vs. T47D^Ctrl^ cells (27.6% vs. 17.9%) (Figure 4D). Relative to T47D^Ctrl^, T47D^403^ also show enrichment of ALDH^high^/CD44^high^/CD24^low^ cells (Figure 4E), which according to literature should have increased tumorigenic and metastatic potential [46]. These molecular features are congruent with acquisition by T47D^403^ cells of cytoskeleton-related molecular changes known to favor cell migration/invasion (scheme in Figure 4F, left, based on global gene/protein expression analyses, not shown), defective actin stress fiber formation (Figure 4F, middle), and increased cell migration (Figure 4F, right). Further, 3D T47D^403^ mature acini, relative to 3D T47D^Ctrl^ acini, display more proliferating CD44^high^ cells (Figure 4G).

These findings indicate that, within a breast cancer cell population, based on the transcriptional functionality of RARA, a higher endogenous RA synthesis favors the proliferation of cells with pro-invasive features via RARA-regulated PI3K/AKT signaling pathway.

Overall, our studies using breast cancer cells let us envision that the different RA biological actions had to do with a cell-autonomous mechanism capable of determining cell fate decisions based on the combinatorial effects of both transcriptional and non-transcriptional RARA functions in response to RA variation. To prove the existence of this mechanism, we needed a developmental model that let us harness precise physiological RA variation to assess the combinatorial effects of spatiotemporal activation of the two RARA functions. The 3D morphogenesis model of non-tumorigenic human mammary epithelial HME1 cells, which express wild type RARA, wild type PI3K subunits, and ADLH1A1 (see Supplementary Materials and Methods), seemed to us a suitable model to test our hypothesis.

### Tracing the different biological actions of physiological RA to a RARA mechanism of mammary epithelial cell fate

**Figure 5:**
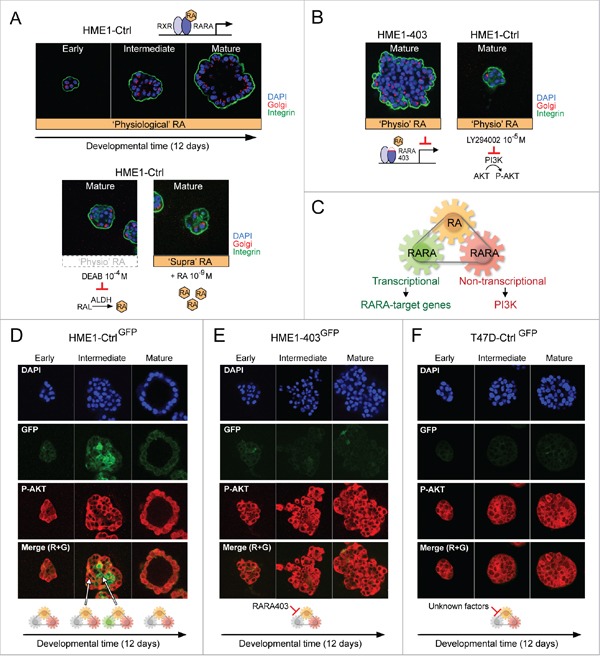
Tracing the different biological actions of physiological RA to a RARA mechanism of mammary epithelial cell fate **A-B**. HME1^Ctrl^ human mammary epithelial cells 3D morphogenesis (A, top) requires precise ‘physiological’ RA variation as well as transcriptional and non-transcriptional RARA functions, because 3D morphogenesis is hampered by both perturbation of ‘physiological’ RA level (e.g. ALDH inhibition by DEAB or addition of exogenous ‘supraphysiological’ RA) (A, bottom) and inhibition of either RARA transcriptional function (B, left) or RARA-PI3K signaling pathway (B, right). **C**. Hypothetical RA-RARA mechanism of mammary epithelial cell fate encompassing a RA metabolic module, a transcriptional RARA module exerting an epigenomic control of RARA-target genes, and a non-transcriptional RARA module controlling PI3K activity. **D**. HME1^Ctrl^ cells stably transfected with RARE-GFP (HME1^Ctrl-GFP^) show differential spatiotemporal activation of RARA transcriptional signaling (GFP) and RARA-PI3K signaling (P-AKT) by ‘physiological’ RA at different stages of 3D morphogenesis. **E-F**. ‘Physiological’ RA fails to induce transcriptional RARA signaling (no GFP), but keeps sustaining RARA-PI3K signaling (P-AKT) during aberrant morphogenesis of both HME1^403-GFP^, with inhibition of RARA transcriptional function by RARA403 (E), and T47D^Ctrl-GFP^, with inhibition of RARA transcriptional function by unknown factors (F).

When seeded in 3D culture on Matrigel, HME1 cells develop into lumen-enclosing monolayers typical of normal breast ducts and lobules in about 12 days (Figure 5A, top). We first set out to test if precise endogenous RA level generated from RA precursors in the 3D culture microenvironment, as well as the integrity of both transcriptional and non-transcriptional RARA functions, were required for normal 3D HME1 morphogenesis. As shown here, perturbation of physiological RA level by either inhibiting ALDH-mediated RA synthesis with DEAB, or adding ‘supraphysiological’ exogenous RA (10^-9^ M), hinders 3D HME1 development (Figure 5A, bottom). We know from our previous studies that inhibition of RARA transcriptional function by genetic factors in HME1 cells [18, 29, 31] results into morphologically aberrant 3D acinar structures (see a representative 3D acinus formed by HME1 expressing the RARA403 dominant negative mutant allele in Figure 5B, left [18]). We instead found, as shown here, that RA promotes RARA-PI3K (p110α) interaction in the HME1 cell context (Supplementary Figure S5B), and that inhibition of PI3K activity hinders 3D acinar development (Figure 5B, right). Overall, these findings let us envision a HME1 cell RA-RARA mechanism encompassing a ‘RA metabolic module’ that generates RA variation and is integrated with both a ‘transcriptional RARA module’, capable of exerting an epigenomic control of RARA-target genes, and a ‘non-transcriptional RARA module’, capable of controlling PI3K activity (Figure 5C).

To assess if physiological RA variation generated during 3D morphogenesis indeed induces the concerted dynamic activation of the two RARA functions, we imaged HME1^Ctrl-GFP^ cells, stably transfected with a RARE-GFP reporter, by confocal microscopy. With this approach, we detected activation of PI3K (assessed as P-AKT, red) at all stages of 3D morphogenesis, and activation of RARA transcriptional function (assessed as GFP, green) only at intermediate stages, in a subset of cells likely destined to clear the lumen (Figure 5D). These observations let us infer that RARA transcriptional function, when inhibited, enables the pro-proliferative and pro-survival actions of ‘physiological’ RA and, when activated, enables RA growth-inhibitory and pro-apoptotic actions. In the latter case, the biological effects of the RARA transcriptional function override the biological effects of the non-transcriptional RARA function. Conversely, at all stages during aberrant 3D morphogenesis of HME1^403-GFP^ cells, with an inhibited RARA transcriptional function, we detected only signs of PI3K activity (assessed as P-AKT) (Figure 5E). Similarly, at all stages during aberrant 3D development of T47D^Ctrl-GFP^ cells, we detected only signs of PI3K activity and RARA transcriptional inactivity (Figure 5F). The latter is likely due to genetic alterations other than RARA mutations, because as mentioned before, T47D cells express wild type RARA. We tested and found that, in the HME1 cells context, genetic alterations that do not affect the RARA structural integrity, but negatively interfere with just the RARA transcriptional function (e.g. mutations of the RA transport protein CRABP2 [29, 47] and ectopic expression of MYC [48]), let physiological RA activate only the RARA-PI3K/AKT signaling pathway (Supplementary Figure S7). Thus, HME1 cell fate decisions during 3D morphogenesis seem to be determined by how effectively the transcriptional component of the RARA mechanism keeps in check the non-transcriptional RARA component in response to ‘physiological’ RA variation.

**Figure 6:**
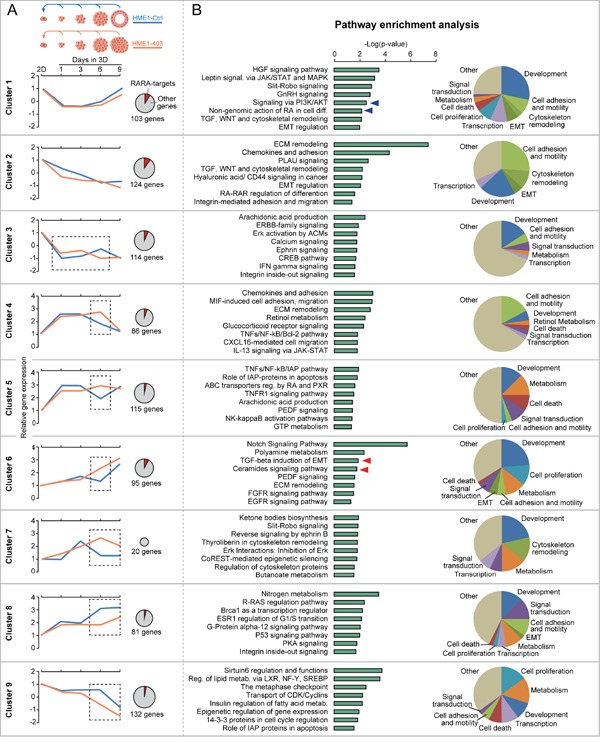
Bioinformatics analysis sheds light on the dynamics of RARA-regulated signaling pathways during 3D mammary epithelial morphogenesis **A**. Comparison of the RNA-seq profile of HME1^Ctrl^ acini at 1, 3, 6, and 9 days of 3D morphogenesis relative to cells grown in 2D culture identifies clusters of genes (including RARA-targets, see red portion of the pie charts) with similar expression trend during 3D morphogenesis (blue lines). The expression of many of these genes is significantly (p<0.05) different at specific stages (dotted squares) of 3D HME1^403^ aberrant morphogenesis (orange lines). **B**. According to Metacore pathway enrichment analysis, these gene clusters are significantly (p<0.05) associated with specific signaling pathways (left) involved in diverse cellular functions (pie charts on the right), including pathways modulated by RARA transcriptional (red arrows) and non-transcriptional (blue arrows) functions. Statistical analysis as reported in Supplementary Materials and Methods.

Next, we asked whether the extent of physiological RA signal variation influences cellular decisions at different 3D developmental stages of HME1^Ctrl^ and HME1^403^ cells by determining combinatorial effects of different signaling pathways. We got a glimpse on the dynamics of signaling pathways activated by physiological RA by bioinformatics analysis of the transcriptome (RNA-seq.) profile of both HME1^Ctrl^ and HME1^403^ at different stages of morphogenesis (Supplementary Figure S8, based on Supplementary Table S2). Interestingly, bioinformatics analysis of HME1^Ctrl^ 3D acini at 1, 3, 6, and 9 days of morphogenesis relative to 2D HME1^Ctrl^ cells with a strategy based on Monocle [49] let us identify nine gene clusters, including subsets of direct RARA-target genes, that share a similar expression trend during 3D morphogenesis (blue lines) (Figure 6A, and Supplementary Table S3). Some of these clusters are associated with morphogenetic signaling pathways known to be either directly transcriptionally regulated by RARA (see the pro-apoptotic SMPD3-ceramide and the TGFB-TGFBR2 signaling pathways of cluster 6, red arrows), or non-transcriptionally regulated by RARA (see PI3K/AKT signaling pathway of cluster 1, blue arrows) (Figure 6B). Moreover, comparison between HME1^403^ and HME1^Ctrl^ at corresponding stages of 3D maturation, let us highlight significant (p<0.05) deregulation of gene expression at stages either preceding lumenogenesis (day 6), or after completion of lumen formation (day 9) (Figure 6A, orange lines). It is noteworthy that bioinformatics analysis of the transcriptome of HME1^403^ vs. HME1^Ctrl^ cells in 2D culture did also reveal that mammary epithelial cells harbor a ‘built-in’ potential to undergo either normal or aberrant 3D morphogenesis even before they are seeded in 3D culture (Supplementary Figure S9).

Overall, confocal imaging analysis and bioinformatics analysis did provide complementary evidence supporting the conclusion that HME1 mammary epithelial cell fate decisions reflect the combinatorial biological effects induced by the spatiotemporal activation of both transcriptional (epigenetic) and non-transcriptional RARA functions by physiological RA. Clearly, the cell context-specific RARA transcriptional epigenetic plasticity that determines morphogenetic processes is critical to deter cell transformation.

## DISCUSSION

All-*trans* RA, the bioactive derivative of Retinol (Vitamin A), a powerful physiological anticancer agent, can paradoxically foster cancer initiation and progression. Resistance to physiological RA growth-inhibitory action was traced for the first time to RARA transcriptional inactivity due to leukemia-associated dominant negative RARA mutations, which determines genome-wide epigenetic deregulation of RARA-target genes [50, 51]. With the exception of leukemia, according to The Cancer Genome Atlas, most cancers, including breast cancer, carry many genetic alterations but not RARA mutations, even if they all display resistance to physiological RA anticancer action. Upon stable inhibition of RARA transcriptional function by ectopic expression of dominant negative RARA mutant alleles in the mouse mammary gland, physiological RA was shown to sustain aberrant branching morphogenesis and excessive cell proliferation via a cell-autonomous mechanism [28]. Similarly, both mouse and human non-tumorigenic mammary epithelial cells, upon functional inhibition of RARA transcriptional activity, develop *in vitro* resistance to the morphogenetic, growth-inhibitory actions of physiological RA that, nevertheless, contributes to promote cell survival/proliferation [18, 33]. Further, as emerged from clinical cancer chemoprevention trials [36, 38, 39], cancer cells can paradoxically grow and invade more in response to low supraphysiological RA treatment, implying that there must be a mechanism that mediates the cancer-promoting action of both physiological and even supraphysiological RA.

In this study we provide evidence that both the anti-cancer and the cancer-promoting action of physiological RA in breast cancer have roots in a developmental RARA epigenetic mechanism of mammary epithelial cell fate. First, by harnessing the response to supraphysiological (exogenous) RA variation of breast cancer cells with a different degree of transcriptional RARA functionality, we found that RA coordinates, in a dose-dependent fashion, both transcriptional RARA signaling a non-transcriptional RARA-PI3K signaling. At each RA concentration, the cancer cell growth outcome seems to reflect the combined effects of both the ‘absence’ of epigenetic transcriptional activation of RARA-targets (e.g. tumor suppressor functions like RARB2 and TGFBR2) and activation of PI3K kinase effectors (e.g. P-AKT).

Second, by using a panel of isogenic HME1 mammary epithelial cells, we found that also physiological (endogenous) RA variation, generated in the course of 3D morphogenesis of lumen-enclosing mammary epithelial cell monolayers, induces the concerted spatiotemporal activation of both a transcriptional signaling and a PI3K signaling via distinct RARA functions. Both precise endogenous physiological RA variation and the integrity of the two (transcriptional and non-transcriptional) RARA functions are indispensable to accomplish the mammary epithelial morphogenetic processes. We also found evidence that the functional plasticity of the RARA transcriptional (epigenetic) component of this mechanism critically determines mammary epithelial cell fate decisions by keeping in check the effects of RARA-regulated PI3K signaling pathways in the right space and at the right time.

By confocal imaging, we could assess that inhibition of RARA transcriptional activity by factors that selectively hamper RARA transcriptional function in mammary epithelial cells, not only impedes the growth-inhibitory/pro-apoptotic action of physiological RA, but also gives the “go ahead” to physiological RA growth-promoting action, leading to malignant cell transformation. Moreover, bioinformatics RNA-seq analysis of the transcriptional dynamics at different stages of 3D morphogenesis of non-tumorigenic mammary epithelial cells shows that physiological RA dynamically modulates gene clusters associated with signaling pathways under the functional control of both transcriptional and non-transcriptional RARA. When RARA transcriptional activity is functionally inhibited, physiological RA activation of growth-promoting signaling pathways, like PI3K/AKT, is no longer counteracted by transcriptional RARA activation of tumor suppressor signaling pathways like the TGFB-TGFBR2 signaling pathway.

Development of strategies suitable to assess the spatiotemporal dynamics of epigenetic events at single cell resolution [52–54], as well as transcriptional/non-transcriptional RA-RARA-regulated signaling networks [55] in mammary epithelial cells, might help us deepen our understanding of the workings of the RARA epigenetic mechanism of mammary epithelial cell fate that, when goes awry, drives breast tumorigenesis.

The HME1 RARA mechanism of mammary epithelial cell fate has features of a proposed mechanism of normal mammary branching morphogenesis. HME1 cells, which are ER+, RARA+, and ALDH1A1+, are capable of forming lumen-enclosing monolayers like mammary epithelial cells found at the branching points of small ducts of the normal human breast epithelium [56]. A non-transcriptional RARA function seems to be involved in excessive mammary ductal branching morphogenesis in female mice with inhibition of RARA transcriptional function by a dominant negative RARA mutant under the murine mammary tumor virus (MMTV) promoter [28]. Moreover, consistent with evidence that in the HME1 cell context PI3K is indispensable for 3D acinar development, targeted homozygous ablation of the PI3K catalytic subunit P110α in transgenic mice severely impairs mammary gland development [57]. In contrast, constitutive activation of PI3K by forced recruitment of P110α to the membrane leads to increased mammary ductal branching and proliferation [58]. PI3K would integrate both mechanical and biochemical signaling of branch initiation and elongation in cultured mouse epithelial cells via its effectors AKT and RAC1 [59]. The mechanism and biology of RA regulation of mammary epithelial cell fate can let us improve detection, prevention, and treatment of early breast cancer.

Factors that negatively affect the RARA transcriptional function are expected to predispose mammary epithelial cells to physiological RA cancer-promoting effects. In breast cancer cells it is not always possible to pinpoint “the” factor(s) that, by inhibiting RARA transcriptional function, make RA exert a cancer-promoting action. Since breast cancer is a disease related to aging [60], not only mutations, but also deterioration of breast tissue *per se* might be a factor capable of negatively affecting the epigenome of breast epithelial cells by weakening the transcriptional functionality of RARA. As shown in this study, in the T47D breast cancer cell context, epigenetic effects due to increased inhibition of RARA transcriptional function result in the expansion of the pool of cells with high RA synthesis (ALDH^high^ cells), including a subset of ALDH^high^/CD24^low^/CD44^high^ cells, with increased stemness, pro-proliferative, and pro-invasive properties [45, 46]. Thus, the breast cancer cell context-specific transcriptional functionality of the RARA mechanism, not only differentially affects the epigenetic state of RARA-target genes, but also determines cancer-promoting effects of physiological RA synthesis due to the level of ALDH activity.

Methods to detect early signs of physiological RA cancer-promoting action in breast tissue could let us identify, and consequently target, insidious effects of physiological RA, thus halting or delaying breast cancer progression. Molecular signs of a dysfunctional RARA mechanism have been detected in breast cancer tissue. DNA hypermethylation of transcriptionally repressed RARA-target genes, which is interpreted as functional inhibition of RARA transcriptional activity [40, 61, 62], or AKT phosphorylation (P-AKT), which is interpreted as aberrant activation of PI3K kinase [63], were found in early stages breast cancer, even before evidence of breast cancer in mammograms [61].

As shown here by bioinformatics RNA-seq. analysis, HME1 mammary epithelial cells have a ‘built-in’ potential to develop into morphologically normal or aberrant 3D structures in the presence of ‘physiological’ RA based on the functional transcriptional status of RARA. Recently, we reported that the protein profiles of HME1 cells that, due to different mutations, are resistant to the morphogenetic (growth-inhibitory/pro-apoptotic) action of physiological RA, differ from normal cells in 2D culture, i.e. before they reveal an aberrant morphology once they develop in 3D culture [31]. One of these protein changes is overexpression of Annexin A8 (ANXA8), a member of the annexin superfamily of membrane- and calcium-binding proteins. Ectopic expression of ANXA8 in the HME1 cell context is sufficient to make cells resistant to physiological RA, with consequent development into 3D amorphous structures resembling ductal carcinoma in situ (DCIS). Remarkably, being ANXA8 significantly overexpressed in DCIS versus normal tissue [31], it could be a valuable biomarker to infer ongoing physiological RA cancer-promoting action in breast tissue samples before breast cancer onset. When signs of physiological RA are detected in breast epithelial cells, epigenetic drugs capable of reawakening RARA-transcriptionally-regulated tumor suppressor activities, in combination with drugs weakening the effect of RA activation of PI3K tumorigenic signaling pathways, could be used to delay breast cancer progression by physiological RA.

**Figure 7:**
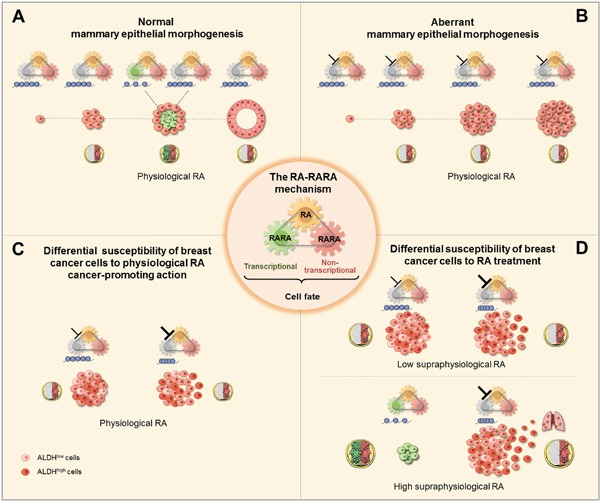
Scheme of the RA-RARA epigenetic mechanism of breast (cancer) epithelial cell fate (center) **A**. Physiological RA acts as the *Janus bifrons* god by regulating, in a spatiotemporal fashion, both the transcriptional RARA epigenetic function (chromatin changes at RARA-target genes, blue) and non-transcriptional RARA function to determine mammary epithelial cell fate decisions necessary for normal morphogenesis. **B**. Physiological RA promotes aberrant morphogenesis of mammary epithelial cells with epigenetic repression of RARA-target genes due to inhibition of RARA transcriptional function (⍔) by activating non-transcriptional RARA. **C**. Differential susceptibility to physiological RA cancer-promoting action between breast cancer cell populations with increasing degree of inhibition of RARA transcriptional epigenetic functionality, mild (⍔) or severe (⍔), depends on the heterogeneity of RA synthesis by ALDH. **D**. Differential susceptibility to either anticancer or cancer-promoting actions of low/high supraphysiological RA treatment also depends on the cell-context specific degree of RARA transcriptional epigenetic functionality.

In summary, the cell context-specific plasticity of the RARA epigenetic mechanism of mammary epithelial cell fate decisions, which is critical to determine different actions of both physiological and supraphysiological RA (see scheme Figure 7), can be harnessed to improve strategies for breast cancer detection, prevention, and treatment.

## MATERIALS AND METHODS

Detailed Materials and Methods are described in the Supplementary Information.

### Cells

T47D, HME1, and MCF7 clonal lines were developed by stable transfection and in part described in previous studies [18, 29, 32, 37]. RARA knock down in T47D^403^ cells was performed by stable transfection with pSuper-shRARA. GFP clonal lines were generated by stable transfection with a 3X-RARE-d2EGFP construct. T47D^403-RFP^ cells were developed by stable transfection of T47D^403^ with pDsRed2-C1. Cell line identity was checked by short tandem repeat (STR) analysis and/or detection of transfected plasmids by PCR.

### Drug treatments

Unless otherwise specified, treatments in 2D culture were performed for 72h. For P-AKT induction, treatments were performed in serum-free medium for 1h on starved cells. Treatments in 3D culture were performed in growth medium plus 2% Matrigel.

### Quantitative RT-PCR

Transcript levels were analyzed by real time PCR with SybrGreen, and quantified by the Delta-deltaCt method using GAPDH for normalization.

### Luciferase assay

Luciferase assays were performed by using Dual Glow Luciferase assay on cells transiently transfected with CAGA-luciferase, using renilla luciferase for normalization.

### Protein analysis

Western blot was performed according to standard protocols using anti-P-AKT(Ser473), anti-RARA, anti-actin, or anti-GAPDH antibodies. Protein bands were quantified with Image J (NIH).

### Analysis of cell proliferation, migration, invasion, and stress fibers

Cell proliferation was assessed by colony formation assay in 2D culture, and EdU incorporation followed by confocal analysis in 3D acini. Cell migration and invasion were assessed by wound assay and Boyden chamber assay, respectively. Actin stress fibers were visualized with rhodamine-conjugated phalloidin.

### Confocal imaging of 3D morphogenesis

Cells grown in three-dimensional (3D) culture on reconstituted basement membrane (Matrigel) were analyzed at different 3D stages by immunocytochemistry followed by confocal microscopy as described [18, 29].

### Global gene expression and bioinformatics analysis

Global gene expression at different 3D stages of HME1 morphogenesis was assessed by RNA-sequencing. Gene clusters were identified based on [49]. Pathway enrichment analysis was performed with Metacore. Global gene expression in T47D cells was assessed by Affymetrix microarrays. RARA-target genes were identified based on [64, 65]. Data were deposited in Gene Expression Omnibus (GEO) (accession number GSE57119).

### In vivo studies

Cells were injected subcutaneously in the flank region or in the mammary fat pad of female athymic NCr-nu/nu mice fed either a control diet or a RA-containing AIN-93G diet. For metastasis analysis, 6 weeks after xenograft tumor removal, mice were euthanized and analyzed for the presence of RFP-positive cells. Experiments were pre-approved by the RPCI Animal Care and Use Committee.

### Statistical analysis

For *in vitro* experiments, the group means were compared by Student's t-test to determine significance. The effect of transcriptional/non-transcriptional RARA signaling pathways on cell growth was assessed by Standard Linear Regression. *In vivo* data were analyzed by one-way ANOVA, followed by multiple comparison tests.

## SUPPLEMENTARY FIGURES AND TABLES








