# Supplementary material for: Tracing anti-cancer and cancer-promoting actions of all-trans retinoic acid in breast cancer to a RARa epigenetic mechanism of mammary epithelial cell fate
Source: Oncotarget. 2016 Nov 22;7(52):87064–80. doi: 10.18632/oncotarget.13500 (PMC5349971; doi:10.18632/oncotarget.13500)
Supplement: Supplementary file 1 [file oncotarget-07-87064-s001.pdf]

Tracing anti-cancer and cancer-promoting actions of all-*trans* retinoic acid in breast cancer to a RARα epigenetic mechanism of mammary epithelial cell fate

SUPPLEMENTARY DATA

SUPPLEMENTARY MATERIALS AND METHODS

Cells and cell culture

For two-dimensional (2D) culture, the breast cancer cell lines MCF7, T47D (both from ATCC, Manassas, VA) and derived clones were cultured in DMEM medium (Life Technologies, Carlsbad, CA) plus 5% fetal bovine serum (Life Technologies). The telomerase-immortalized, non-tumorigenic human mammary epithelial cell line h-TERT-HME1 (here referred to as HME1) (Clontech, Mountain View, CA) and derived clones were grown in Mammary Epithelial Growth Medium (MEGM) as per manufacturer's instructions (Lonza, Walkersville, MD). For three-dimensional (3D) culture, HME1- and T47D-derived clones were grown at low density (3-5×10^3 single cells/well) on 8-well chamber slides coated with growth-factor reduced Matrigel (BD Biosciences, San Jose, CA) in growth medium plus 2% Matrigel for 10-12 days, refreshing the medium every 2-3 days.

The T47D-derived clones $T47D^{403}$ (formerly named DNC8) carrying the human dominant-negative RARA403 mutant [1], and $T47D^{Ctrl}$ (formerly named LXC5), carrying the cognate empty vector, were previously described [2]. To develop the $T47D^{G303E}$ clone, the RARA mutant RARAG303E (kindly provided by Dr. A. Kakizuka, Kyoto University) [3] was subcloned into pLXSN and stably transfected into T47D by using Lipofectamine LTX (Life Technologies). After selection with 1 mg/ml G418, positive clones were isolated and expanded. To develop MCF7 stably expressing RARA403 and the relative control line, cells were transfected with either pLXSN-RARA403 [1] or the cognate empty vector by using Lipofectamine LTX (Life Technologies), and selected with 2 mg/ml G418. To knock down PPARD or RARA in $T47D^{403}$ cells, shRNA sequences targeting either PPARD (5'- GAT TCA GAA GAA GAA CCG C - 3') or RARA (5'- GCC TCT CAT CCA GGA AAT G - 3') were cloned into the pSuper-retro vector (Oligoengine, Seattle, WA), according to the manufacturer's instructions and stably transfected into $T47D^{403}$ cells by using Lipofectamine LTX. As a control, $T47D^{403}$ cells were stably transfected with pSuper-retro vector carrying a control scrambled sequence (pSuper-shSCR) [4]. After selection with 1 μg/ml puromycin, single clones were isolated, expanded and tested for PPARD or RARA knock down by Western Blot. To develop $T47D^{403\text{-}RFP}$ cells, stably expressing RFP, $T47D^{403}$ were transfected with pDsRed2-C1 (Clontech). To develop $T47D^{TR2DN}$ clones, stably expressing a TGFBR2 dominant negative, the TGFBR2 cDNA portion encoding the TGFBR2 extracellular domain (aa. 1-159) [5] was amplified with primers introducing a stop codon after aminoacid 159 (sense: 5' - CAC CAT GGG TCG GGG GCT GCT CA - 3' and antisense: 5' - CTA GTC AGG ATT GCT GGT GTT ATA T - 3'), cloned into pcDNA3.1-V5-His-TOPO (Life Technologies), and stably transfected into $T47D^{Ctrl}$ cells. To develop $T47D^{403}$ clones overexpressing TGFBR2, the TGFBR2 cDNA was amplified with primers sense: 5'- GGC TCG GTC TAT GAC GAG CAG-3' and antisense: 5'- TCC TGC TGC CTA TTT GGT AGT G-3', cloned into pcDNA3.1-V5-His-TOPO (Life Technologies), and stably transfected into $T47D^{403}$ cells. $HME1^{403}$, stably expressing the RARA mutant RARA403 (formerly named DNC4), $HME1^{KRK}$, stably expressing the CRABP2 K20A/R29A/K30A (KRK) mutant [6], and $HME1^{MYC}$, stably overexpressing MYC, were previously described [4, 7, 8]. GFP-positive clones were developed by stable transfection with a 3X-RARE-d2EGFP construct developed by substituting the EGFP portion of 3X-RARE-EGFP (kindly provided by Dr. L. Chen, Stanford, CA) with destabilized d2EGFP from pd2EGFP-1 (Addgene, Cambridge, MA). This destabilized GFP, with a half-life of just 2 hours, was specifically chosen to monitor rapid expression changes in response to RA variation. Stable clones were expanded, analyzed by PCR for the presence of the construct, and screened for expression of d2EGFP in response to RA (10^{-6} M, 24h).

HME1, T47D and MCF7 express wild type RARA1. HME1 cells express ALDH1A1, as per immunocytochemistry analysis (data not shown). Exon sequencing of the genes encoding the two PI3K subunits (*PIK3CA* and *PIK3R1*) confirmed the presence of the *PIK3CA* H1047R activating mutation in T47D cells, while it did not identify any deleterious mutations in HME1 cells (data not shown). The identity of clones and cell lines was checked by STR analysis and/or detection of transfected plasmids by PCR.

Drugs and treatments

All-trans retinoic acid (RA) (Sigma, St Louis, MO), the RARA agonist AM580 (Enzo Life Sciences, Farmingdale, NY), the PI3K inhibitor LY294002 (Enzo

Life Sciences), the ALDH inhibitor DEAB (Sigma), the RARA antagonist ER50891 (kindly provided by Dr. Kikuchi, Discovery Research Laboratories, Ibaraki, Japan), the PPARD agonist GW501516 (Enzo Life Sciences), and ROH (Sigma) were dissolved in ethanol. The DNA methylation inhibitor 5-aza-2′-deoxycitidine (5-Aza) (Sigma) was dissolved in 0.45% NaCl containing 10 mM sodium phosphate (pH 6.8). TGFB1 (R&D Systems, Minneapolis, MN) was dissolved in 4 mM HCl + 0.1% BSA. Drugs were stored in the dark at -80°C. The concentration of DEAB used for treatment (10^{-4} M) was based on reference [9] and did not show any signs of toxicity by LIVE/DEAD cell viability assay (Life Technologies) (data not shown). Unless otherwise specified, treatments of T47D-derived clones in 2D culture were performed for 72h, replacing the drug-containing medium every 24h. For co-treatments with LY294002, cells were pre-incubated with LY294002 for 1h prior to the addition of RA or AM580. For P-AKT induction, co-immunoprecipitation, and PLA experiments on T47D-derived clones, treatments were performed in serum-free medium for 1h on cells previously starved in serum-free medium for 24-72h. Treatments in 3D culture were performed in growth medium plus 2% Matrigel, and the drug-containing medium was refreshed every 1-2 days for the duration of the experiment. All drug treatments were performed under dim yellow light.

Note that in *in vitro* experiments we used the term 'supraphysiological' and 'physiological' RA/ROH when cells were grown with or without addition of exogenous RA/ROH, respectively.

Luciferase assay

Cells grown in a 24-well plate were transfected with 10 ng pRL-TK and 300 ng of either pGL3-$(CAGA)_9$-TK-luc, containing the luciferase gene under the control of nine TGFB-inducible CAGA boxes (kindly provided by Dr. Andrei Bakin, Roswell Park Cancer Institute, Buffalo, NY) [10], pGL3- PPRE-luc, containing the luciferase gene under the control of the PPRE response element (kindly provided by Dr. Noa Noy, Case Western University, Cleveland, OH), or pGL2-RARB2-luc, carrying the luciferase gene under the control of the RARE-containing RARB2 promoter (kindly provided by Dr. Keiko Ozato, National Institutes of Health, Bethesda, MD) by using Lipofectamine LTX (Life Technologies). After overnight transfection, cells were treated with the indicated drugs for 24–72h. Luciferase activity was measured by using Dual Glow Luciferase Assay System (Promega, Madison, WI) as per manufacturer's instructions.

Quantitative real time RT-PCR (qRT-PCR)

Total RNA obtained with the single-step method using Trizol (Life Technologies) was treated with DNase I (Life Technologies), retrotranscribed into cDNA with High Capacity cDNA RT kit (Life Technologies), and used for real time PCR. Real time PCR was performed on an iCycler (Bio-Rad, Hercules, CA) using the iQ SYBR Green Supermix (Bio-Rad) with primers specific for RARB2 (sense: 5'-GAC TGT ATG GAT GTT CTG TCA G-3'; antisense: 5'-ATT TGT CCT GGC AGA CGA AGC A-3'), CYP26A1 (sense: 5′- GCA ATC TTC AAC CGA ACT CC-3′; antisense: 5′- CTC CTT AAT AAC ACA CCC GAT G-3′), TGFBR2 (sense: 5'- CGC ACG TTC AGA AGT CGG TTA - 3'; antisense: 5'- GGG TCA TGG CAA ACT GTC TCT - 3'), RARRES1 (sense: 3' - AGA CAA CAA GAG GAT TAC CTG C - 5'; antisense: 3' - CTG TGC CAA GTA GTA GTG TGA C - 5'), SMPD3 (sense: 5′-CAA CAA GTG TAA CGA CGA TGC C-3′; antisense: 5′-CGA TTC TTT GGT CCT GAG GTG T-3′), ADRP (sense: 5'-TGA GAT GGC AGA GAA CGG TGT G-3'; antisense: 5'-GGC ATT GGC AAC AAT CTG A-3'), GAPDH (sense: 5'-GAA GGT GAA GGT CGG AGT C-3', antisense: 5'-GAA GAT GGT GAT GGG ATT TC-3'), mouse Cyp26a1 (sense 5'-GAA CCT TAT ACA CGC GCG CAT-3', antisense 5'-CTC TGT TGA CGA TTG TTT TAG TG-5'), or mouse gamma-Actin (sense 5'-GCC GGC TTA CAC TGC GCT TCT T-3', antisense 5'-TTC TGG CCC ATG CCC ACC AT-3'). Gene transcript levels were quantified by the Delta-delta Ct method, using GAPDH or mouse gamma-Actin for normalization.

Quantitative chromatin immunoprecipitation (qChIP)

ChIP was performed using reagents purchased from Millipore, following the manufacturer's protocol. RARA occupancy at the putative/known RARA-target gene promoters was assessed by using anti-RARA C-20X antibody (Santa Cruz Biotechnology, Santa Cruz, CA), and histone modifications were assessed by using anti-Acetyl Histone H4 (AcH4) and anti-Histone H3 lysine 4 tri-methylation (3M-H3K4) antibodies (Millipore). Control ChIPs were performed with non-specific IgGs (Millipore). The immunoprecipitated DNA was amplified by real-time PCR with primers specific for the RARE-containing regions and/or the transcription start site (TSS) of RARB2 (sense: 5' - GGT TCA CCG AAA GTT CAC TCG CAT - 3'; antisense: 5' - CAG GCT TGC TCG GCC AAT CCA - 3'), TGFBR2 (sense: 5' – AGC ACC TAG GAG CAA TCT GAA G - 3'; antisense: 5' - CTA GCT CTC TCG TAG CTG CCA - 3'), SMPD3 (sense: 5' - CAG AAG GCT GTG CGA AAC TC - 3'; antisense: 5' - CTC AGA CTC AGC ACC TGG AG - 3'), RARRES1 (RARE) (sense: 5' - ATG CCT CAC CCC GCT GGA ATC - 3'; antisense: 5' – CAG CAA GGC AGG GTA AGA TAA G - 3', for ChIP with anti-RARA), RARRES1 (TSS) (sense: 5' - GAT GCC GCA TCC TAG CAC TAA G - 3'; antisense: 5' – AGG GCG AAG GTC TGT AGC GAG - 3', for ChIP

with anti-AcH4 and anti-3M-H3K4). The DNA relative enrichment was calculated by using the Delta-delta Ct method. The PCR signals obtained for each gene region were normalized to the PCR signal obtained from the input DNA (total chromatin fraction).

Western blotting and co-immunoprecipitation

For RARA detection, sub-confluent cells were grown in standard medium, while for P-AKT detection cells were starved for 72h in serum-free DMEM and treated with or without RA for 1h before lysis with RIPA buffer (50 mM Tris-HCl pH 8.0, 150 mM NaCl, 1% Nonidet P40, 0.1% SDS, supplemented with freshly-added Complete protease inhibitor cocktail (Roche, Branchburg, NJ), 10 mM NaF, 1mM Na_3VO_4). Western blot on total cell lysates was performed according to standard protocols using anti-RARA C-20 (Santa Cruz Biotechnologies) anti-P-AKT(Ser473), anti-GAPDH, or anti-actin (Santa Cruz Biotechnology) antibodies, followed by appropriate HRP-conjugated secondary antibodies (GE Healthcare, Piscataway, NJ) and ECL detection (GE Healthcare). Non-saturated protein bands were quantified by using Image J (NIH).

Co-immunoprecipitation was performed on cell lysates prepared as described above by using Trueblot IP kit (eBioscience, San Diego, CA). Briefly, cell lysates were pre-cleared with TrueBlot anti-rabbit Ig IP beads and incubated over night at 4 °C with either a rabbit anti-p85α antibody (Millipore, Billerica, MA) or a non-specific rabbit IgG (Millipore) as a control. The immunocomplexes were isolated by incubation with TrueBlot anti-rabbit Ig IP followed by centrifugation, extensive washing, and elution with Laemmli loading buffer. Samples were analyzed by Western blot with rabbit anti-p85α, rabbit anti-p110α (Cell Signaling), or rabbit anti-RARA C20 (Santa Cruz Biotechnology), followed by incubation with HRP-conjugated Rabbit IgG TrueBlot and ECL detection.

Proximity ligation assay (PLA)

Subconfluent cells grown in 8-well chamber slides and treated as described above were fixed with 3.7% formaldehyde for 7 min., permeabilized with PBS plus 0.1% Triton X100 for 7 min., blocked with PBS containing 1% BSA, 1% FBS and 0.05% Tween 20 for 2 hours, and incubated with the primary antibodies for 2 hours. Cells were incubated with mouse anti-RARA C-terminus (Millipore, 04-1545) and either rabbit anti-p85 (Millipore, 06-195) or rabbit anti-p110α (Cell Signaling, 4249). Negative controls were incubated with either anti-RARA alone or no antibody. After antibody incubation, PLA was performed in 30 µl droplet reactions by using Duolink in situ PLA kit (Olink Biosciences, Uppsala, Sweden) as per manufacturer's instructions. After counterstaining with DAPI, at least 5 random fields per samples were analyzed by fluorescence microscopy at high magnification. The number and the area of the PLA signals, visible as fluorescent spots, were measured by using Image J and expressed relatively to the number of cells (nuclei) in each field. Both measurements gave similar results, and in both cases the PLA signal from the negative controls was significantly lower than the signal from the experimental samples.

Cell proliferation assays

For 2D cultures, cells were seeded at low density (2.5×10^2 cells/well in 12-well plates) in four replicates, treated with the indicated drugs, and let grow in drug-free medium until discrete colonies were visible. To evaluate cell number, colonies were fixed with methanol and stained with Giemsa (Sigma), and the total colony area was measured by using Image J (NIH). Cell number was also evaluated by MTT vital staining with comparable results (not shown). For 3D culture, cell proliferation was assessed by measuring 5-ethynyl-2'-deoxyuridine (EdU) incorporation with Click-iT EdU imaging kit (Life Technologies), as per manufacturer's instructions. Briefly, cells were grown in 3D culture as described above for 10-12 days, incubated with 40 µM EdU for 2 hours under standard growth conditions, fixed in 3.7% formaldehyde for 20 min., permeabilized with PBS plus 0.5 % Triton X100 for 20 min., washed with PBS containing 3% BSA, reacted with Click-iT reaction cocktail (containing azide-conjugated Alexa Fluor 488 or 594) for 45', counterstained with DAPI, mounted with Vectashield (Vector Laboratories), and analyzed by confocal microscopy.

Cell migration assay (wound healing assay)

Confluent cells, seeded in triplicate in 6-well plates, were scratched with a pipette tip, washed and let grow for up to 48 h. Wound closure was monitored by taking pictures every 24 h in the same position and quantified by comparing the wound surface at the different time points vs the wound surface at time t=0. The wound surface was measured by using Image J (NIH).

Cell invasion assay (Boyden chamber assay)

Cell invasion through Matrigel was tested by using 8 µm BioCoat Matrigel Matrix Invasion Chambers (BD Biosciences) according to the vendor's instructions. The cells, seeded in triplicate onto the upper chamber containing serum-free DMEM, were treated as indicated in the Results section and let invade, through the Matrigel layer, into the lower chamber, containing DMEM + 10% FBS. After 72 h, the cells able to invade were fixed and stained with Giemsa. The area covered by the invading

cells in five random fields per chamber was measured with Image J (NIH).

Actin stress fiber analysis

Sub-confluent cells were fixed with 4% paraformaldehyde, permeabilized with PBS containing 0.1% Triton X100, blocked with PBS containing 1% BSA, 1% goat serum, and 0.05% Tween 20, and stained for actin fibers by using rhodamine-conjugated phalloidin (Life Technologies). Nuclei were counterstained with DAPI. Stained cells were mounted with Vectashield (Vector Laboratories, Burlingame, CA), and analyzed by fluorescence microscopy.

Cytofluorimetric analysis

To assess ALDH activity and CD24/CD44 expression, cells were incubated with Aldefluor (StemCell Technologies, Vancouver, Canada) in the presence or absence of DEAB (background control) according to the manufatcurer’s instructions, then incubated with anti-CD24-PE and anti-CD44-APC (BD Biosciences) in Aldefluor buffer for 30 min. on ice, washed, resuspended in Aldefluor buffer and analyzed on a LSR II flow cytometer (BD Biosciences). The percentage of cells in $CD44^{high}/CD24^{low}$ quadrant and the percentage of $ALDH^{high}$ cells in this subpopulation were measured by using Winlist. To assess cell proliferation concomitantly with ALDH activity and AKT phosphorylation, cells were first labeled with the membrane linker PKH26 (Sigma) according to the manufacturer’s instructions. Briefly, a single-cell suspension containing $5X10^6$ cells in 250 µl Buffer C were stained by addition of 250 µl Buffer C containing 10 µl PKH26 solution. After a 5 min incubation at room temperature, the staining was blocked with 500 µl FBS and 1 ml growth medium. Cells were washed three times with growth medium, then divided in two aliquots. One aliquot was fixed in 2% formaldehyde and used both to check for homogenous staining by fluorescence microscopy, and as time t=0 to evaluate initial PKH26 signal intensity. The second aliquot was seeded back in culture and grown for 72h before assessment of P-AKT level or ALDH activity. To measure P-AKT level, PKH26-labeled and non-labeled single-cell suspensions were washed with PBS, fixed with 2% formaldehyde for 10 min., washed with PBS, blocked with PBS + 1% BSA for 30 min., incubated with anti-P-AKT(Ser473) (Cell Signaling) for 30 min., washed, incubated with Alexa Fluor 488 anti-rabbit (Life Technologies) for 30 min., washed, and analyzed by flow cytometry. ALDH activity was measured by Aldefluor staining as described above. PKH26, P-AKT and Aldefluor intensities were evaluated on a LSR II flow cytometer (BD Biosciences) and analyzed with Winlist. Cells were first gated according to the PKH26 signal in $PKH26^{low}$ (5% fastest-proliferating cells) and $PKH26^{high}$ (5% slowest-proliferating cells), then the single gates were analyzed either for P-AKT level or for ALDH activity.

Confocal analysis of 3D mammary epithelial morphogenesis

3D acini at early, intermediate, or mature stages were immunostained as we previously described [7]. Briefly, cells were fixed with 3.7% formaldehyde for 15 min, permeabilized with PBS plus 0.2% Triton X100 for 15 min, blocked with PBS + 1% BSA, 1% FBS, and 0.05% Tween 20 for 1h, incubated with the primary antibody overnight at 4 °C, washed, incubated with the appropriate fluorescent secondary antibody for 2h at room temperature, washed, counterstained with 300 nM DAPI (Sigma), and mounted with Vectashield (Vector Laboratories). Integrin was immunostained with anti-CD49f antibody (EMD Millipore, Billerica, MA) followed by anti-rat Alexa Fluor 488 (Life Technologies), the Golgi apparatus was detected with anti-GM130 antibody (BD Biosciences) followed by anti-mouse Alexa Fluor 546 (Life Technologies), and P-AKT was detected with anti-P-AKT(Ser473) (Cell Signaling) followed by anti-rabbit Alexa Fluor 546 (Life Technologies). 3D acini were imaged by confocal microscopy.

Confocal analysis of EdU incorporation and CD44 expression in 3D acini

Acini grown in 3D culture for 10-12 days were incubated with EdU and stained with Click-iT EdU imaging kit (Life Technologies) as described above. EdU-stained acini were incubated with anti-CD44-APC (BD Biosciences) overnight at 4 °C, washed, incubated with anti-rabbit Alexa Fluor 488 (Life Technologies) for 2 hours at room temperature (to detect CD44 with a green fluorochrome), washed, counterstained with DAPI, and mounted with Vectashield (Vector Laboratories). 3D acini were imaged by confocal microscopy and analyzed blindly. The number of EdU-positive and the $CD44^{high}$ cells in each acinus was assessed relative to the total number of cells (measured based on DAPI staining).

Microarray analysis

Total RNA was extracted with RNeasy mini kit (Qiagen, Germantown, MD) from $T47D^{Ctrl}$ and $T47D^{403}$ cells treated with or without high supraphysiological RA (10^{-6} M, 72h) and treated with DNase (Qiagen). The quality of the RNA was tested on an agarose gel containing 1% formaldehyde and by measuring the ratio between absorbance at 260 and 280 nm. Double stranded cDNA was generated from 5 mg of RNA using Superscript ds

cDNA synthesis kit (Life Technologies) and T7-oligo(dT) primers. The cDNA was purified with GeneChip Sample Cleanup Module (Affymetrix, Santa Clara, CA) and used to synthesized biotin-labeled cRNA with Enzo RNA transcript Labeling Kit (Enzo Life Sciences). Purified cRNA was quantified by spectrophotometric methods and the concentration was adjusted in order to exclude the carryover of unlabeled RNA. 11mg of cRNA were then fragmented in fragmentation buffer (Affymetrix) at 95 °C for 35 min. and hybridized for 16h at 45 °C onto U133A microarrays (Affymetrix). After washing and staining, the chips were scanned in a Hewett-Packard/Affymetrix scanner at 570 nm. For all the experiments the 5'/3' ratios of GAPDH were 0.7- 0.9. In comparison experiments scaling factor, noise and presence calls were similar. Array data files were processed using the MAS5.0 algorithm in the *affy* R library within Bioconductor suite to generate expression summary values for each probe set. MAS5.0 based "present calls" was used to filter out probe sets whose expression intensities were close to background noise across all samples (that is, without "present" status in all samples including both treatments and controls), and probe sets whose "present calls" was inconsistent within the biological replicates in any of the four conditions. Further quality control included the usage of latest *affy* annotation package to filter out probes sets without any known gene assignment. The *Limma* program in the *Bioconductor* package under R computing environment was used to calculate the level of differential gene expression. Briefly, a linear model was fit to the data (*i.e.*, two-factor design with cell means corresponding to the different conditions and a random effect for array). A list of differentially expressed genes constrained by p-value < 0.05 and absolute fold change > 2 was obtained for each comparison. Within this list, RARA-target genes were first identified based on published lists of genes known to be direct RARA-targets [11] and/or to be bound by RARA in breast cancer cells [12]. The expression levels of these RARA-targets were mean-centered and visualized in a heat map. The microarray data were deposited in GEO (accession number GSE57119).

RNA-seq analysis

Total RNA was extracted from cells grown in 2D culture or from 3D acini at different stages by using Trizol and purified on Zymo columns (Zymo Research, Irvine, CA) according to the manufacturer's instructions. RNA libraries were prepared by using Encore Complete RNA-Seq Library Systems (NuGen, San Carlos, CA) and run on a HiSeq2000 sequencing system (Illumina, San Diego, CA). After samples were de-multiplexed with fastq-multx, reads were aligned with TopHat [13] using UCSC hg19 as the reference genome. The genome sequence and annotations were downloaded from iGenomes (Illumina), and a transcriptome index was built for the given annotation by using Bowtie. After mapping the reads to the reference genome, the aligned reads were processed by Cufflink and merged together with Cuffmerge. Gene expression levels were estimated with Cuffquant, and differential analysis between groups was performed by using Cuffdiff. For the analysis of expression trend clusters, the transcripts differentially expressed between $HME1^{Ctrl}/HME1^{403}$ 3D acini at different stages and $HME1^{Ctrl}$ grown in 2D culture ($q<0.25$, absolute fold change > 2) were group into clusters with similar expression trend based on [14]. Specifically, the partitioning around medioids (PAM) method was used to group the transcripts based on their expression trend. The similarity between two transcript trends was measured by the scale-independent Pearson correlation between two transcript expression profiles, and the number of distinct clusters was determined by assessing the quality of the clustering results quantified by silhouette width. RARA-target genes within the clusters were identified based on [11, 12]. Pathway enrichment analysis of each cluster was performed by using Metacore (Thomson Reuters).

In vivo studies

All experiments described here were approved by the Animal Care and Use Committee of Roswell Park Cancer Institute (Buffalo, NY). Female athymic NCr-nu/nu mice (6-8 weeks old) were bought from NCI-Frederick Animal Production Program (NCI, Frederick, MD). All mice were kept in a temperature-controlled room on a 12/12-h light/dark schedule, with food and water ad libitum. Two days before injecting the cells, mice were estrogenized by intramuscular injection of Depo-estradiol (Florida Infusion Co, Palm Harbor, FL) at 1.5 mg/kg body weight. For dorsal flank xenografts, mice were subcutaneously inoculated in the flank region (bilateral) with either 5 x 10^6 $T47D^{Ctrl}$ cells or 5 x 10^6 $T47D^{403}$ cells previously resuspended in serum-free DMEM (Life Technologies) and mixed with Matrigel (BD Biosciences) (1:1) in a final volume of 0.2 ml. Mice were randomly divided into groups fed either a control AIN-93G diet or the same diet supplemented with RA (10 mg/kg diet) or ROH (50 mg/Kg diet) for at least 6 weeks (all diets were from Research Diets Inc., New Brunswick, NJ). Tumors size was measured with a digital caliper twice a week, and tumor volumes were calculated as described [15]. At the end of the experiment, mice were euthanized, and tumors and livers were collected for further studies. For metastasis analysis, $T47D^{403}$ cells were first stably transfected with pDsRed2-C1 to obtain $T47D^{403\text{-}RFP}$ clones. A $T47D^{403\text{-}RFP}$ clone highly expressing RFP expression and able to invade *in vitro*, was xenografted into nude mice fed a control diet or a RA-enriched diet. Tumors were surgically removed after 6 weeks or when their size reached 1 cm. Six weeks

after tumor removal mice were euthanized and analyzed for the presence of metastases. RFP-positive cells were detected either by whole body imaging or imaging of single organs by using a Fluorescent Light Box (Lightools Research, Encinitas, CA).

Statistical analysis

For *in vitro* studies, the group means were compared by Student's t-test with Excel or GraphPad Prism 6 to determine significance. Standard linear regression was used to model log-scale growth. In the latter case, numbers reported are exponentiated coefficients and confidence intervals (CI), and should be interpreted multiplicatively. As regressors, dose was considered on the log scale and expression on the linear scale. For *in vivo* studies, data were analyzed by one-way ANOVA, followed by multiple comparison tests (STATISTICA program, Tulsa, OK, USA); all statistical tests were two-sided.

REFERENCES

1. Tsai S, Bartelmez S, Heyman R, Damm K, Evans R and Collins SJ. A mutated retinoic acid receptor-alpha exhibiting dominant-negative activity alters the lineage development of a multipotent hematopoietic cell line. Genes Dev. 1992; 6:2258-2269.
2. Ren M, Pozzi S, Bistulfi G, Somenzi G, Rossetti S and Sacchi N. Impaired retinoic acid (RA) signal leads to RARbeta2 epigenetic silencing and RA resistance. Mol Cell Biol. 2005; 25:10591-10603.
3. Saitou M, Narumiya S and Kakizuka A. Alteration of a single amino acid residue in retinoic acid receptor causes dominant-negative phenotype. J Biol Chem. 1994; 269:19101-19107.
4. Bistulfi G, Pozzi S, Ren M, Rossetti S and Sacchi N. A repressive epigenetic domino effect confers susceptibility to breast epithelial cell transformation: implications for predicting breast cancer risk. Cancer Res. 2006; 66:10308-10314.
5. Tsang ML, Zhou L, Zheng BL, Wenker J, Fransen G, Humphrey J, Smith JM, O'Connor-McCourt M, Lucas R and Weatherbee JA. Characterization of recombinant soluble human transforming growth factor-beta receptor type II (rhTGF-beta sRII). Cytokine. 1995; 7:389-397.
6. Sessler RJ and Noy N. A ligand-activated nuclear localization signal in cellular retinoic acid binding protein-II. Mol Cell. 2005; 18:343-353.
7. Corlazzoli F, Rossetti S, Bistulfi G, Ren M and Sacchi N. Derangement of a factor upstream of RARalpha triggers the repression of a pleiotropic epigenetic network. PLoS One. 2009; 4:e4305.
8. Rossetti S, Hoogeveen AT, Esposito J and Sacchi N. Loss of MTG16a (CBFA2T3), a novel rDNA repressor, leads to increased ribogenesis and disruption of breast acinar morphogenesis. J Cell Mol Med. 2010; 14:1358-1370.
9. Chute JP, Muramoto GG, Whitesides J, Colvin M, Safi R, Chao NJ and McDonnell DP. Inhibition of aldehyde dehydrogenase and retinoid signaling induces the expansion of human hematopoietic stem cells. Proc Natl Acad Sci U S A. 2006; 103:11707-11712.
10. Dennler S, Itoh S, Vivien D, ten Dijke P, Huet S and Gauthier JM. Direct binding of Smad3 and Smad4 to critical TGF beta-inducible elements in the promoter of human plasminogen activator inhibitor-type 1 gene. Embo J. 1998; 17:3091-3100.
11. Balmer JE and Blomhoff R. Gene expression regulation by retinoic acid. J Lipid Res. 2002; 43:1773-1808.
12. Hua S, Kittler R and White KP. Genomic antagonism between retinoic acid and estrogen signaling in breast cancer. Cell. 2009; 137:1259-1271.
13. Trapnell C, Roberts A, Goff L, Pertea G, Kim D, Kelley DR, Pimentel H, Salzberg SL, Rinn JL and Pachter L. Differential gene and transcript expression analysis of RNA-seq experiments with TopHat and Cufflinks. Nat Protoc. 2012; 7:562-578.
14. Trapnell C, Cacchiarelli D, Grimsby J, Pokharel P, Li S, Morse M, Lennon NJ, Livak KJ, Mikkelsen TS and Rinn JL. The dynamics and regulators of cell fate decisions are revealed by pseudotemporal ordering of single cells. Nat Biotechnol. 2014; 32:381-386.
15. Sirchia SM, Ren M, Pili R, Sironi E, Somenzi G, Ghidoni R, Toma S, Nicolo G and Sacchi N. Endogenous reactivation of the RARbeta2 tumor suppressor gene epigenetically silenced in breast cancer. Cancer Res. 2002; 62:2455-2461.

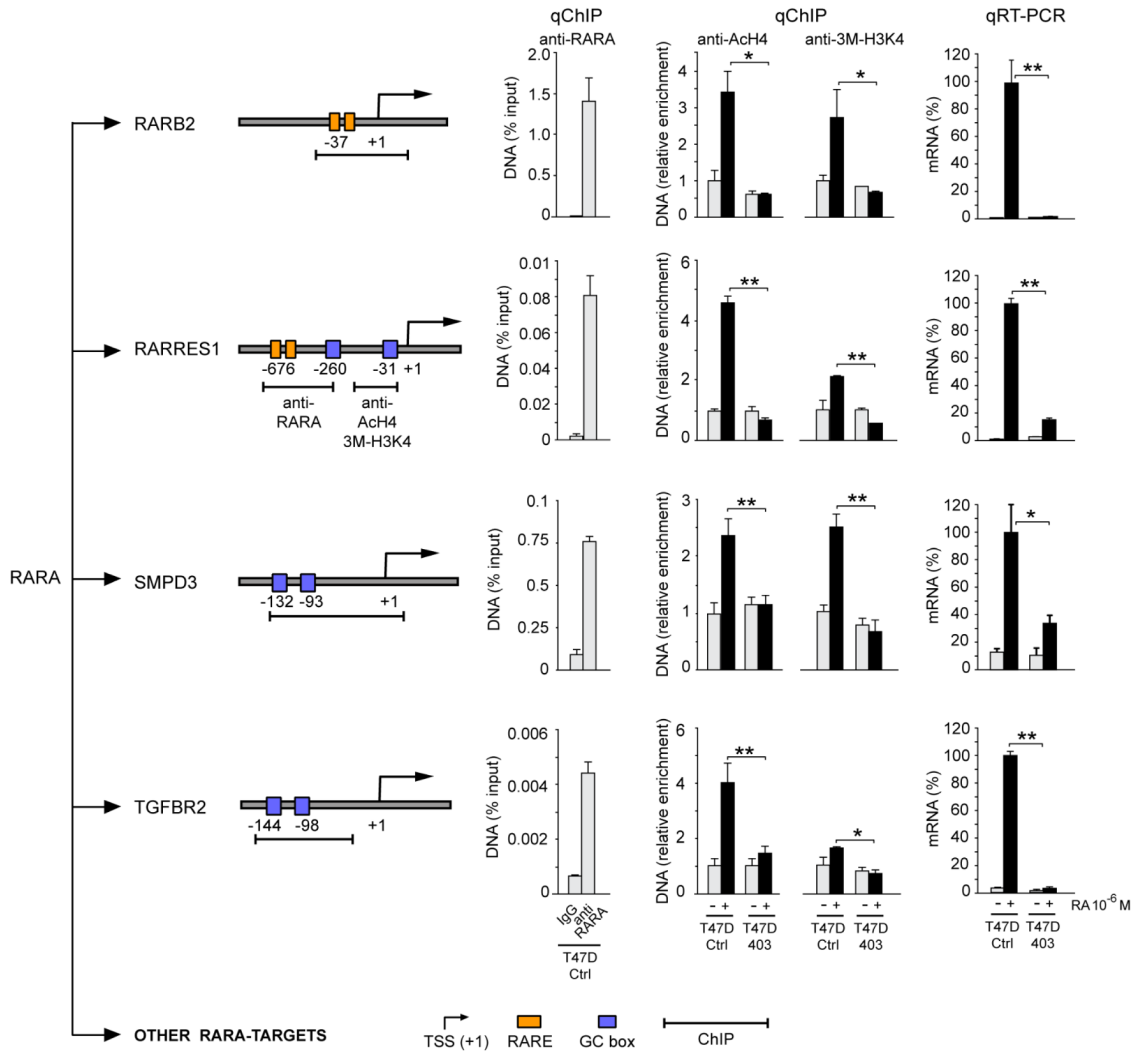

Supplementary Figure S1: Evidence of increased epigenetic repression of tumor suppressor RARA-target genes in the T47D cell context. RARA-target genes (see ChIP with anti-RARA) with tumor suppressor functions are epigenetically repressed in both T47D^{Ctrl} and T47D^{403} under 'physiological' RA conditions, but they can be transcriptionally reactivated by high 'supraphysiological' RA (10^{-6} M) only in T47D^{Ctrl} (see qRT-PCR). Lack of gene reactivation in T47D^{403} cells is associated with repressive histone changes unresponsive to high 'supraphysiological' RA (see qChIP analysis of acetylated histone H4 and tri-methylated histone H3 lysine 4) (*p<0.05, **p<0.01). Significance calculated by Student's t-test.

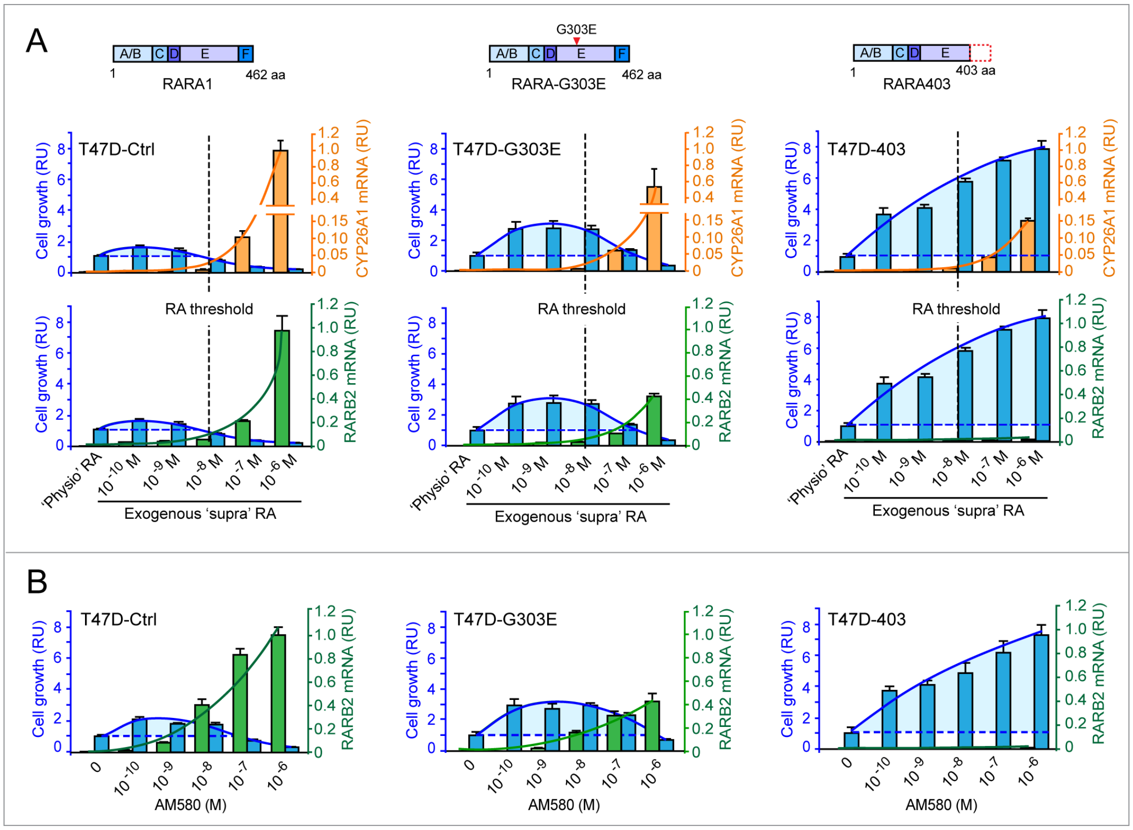

Supplementary Figure S2: Dose-dependent RA effects on cell growth depends on the cancer cell context-specific RARA transcriptional functionality. A. Analysis of CYP26A1 (yellow) and RARB2 (green) transcript levels shows that the transcriptional response of T47D^{G303E} cells, expressing the dominant negative RARAG303E mutant, to RA variation (from 'physiological' to high exogenous 'supraphysiological') determines the cell growth outcome significantly more (**p<0.01) than in T47D^{Ctrl} (expressing wild type RARA) at RA concentrations below the T47D^{Ctrl} threshold (10^{-8} M), and significantly less than in T47D^{403} (expressing the dominant negative RARA403) at concentrations both below (**p<0.01) and above (***p<0.001) the T47D^{Ctrl} threshold. **B.** The RARA agonist AM580 recapitulates the effects of RA on both RARB2 transcription and cell growth. Significance calculated by Student's t-test. (Note: the T47D^{Ctrl} and T47D^{403} panels, from Figure 1, are shown here for comparison with T47D^{G303E}).

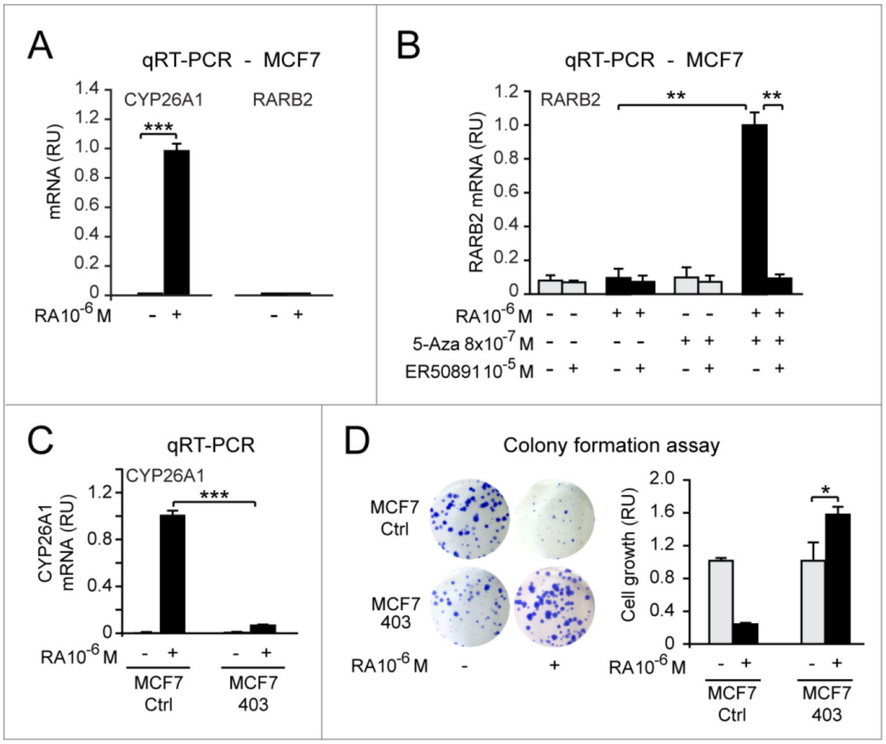

Supplementary Figure S3: Increasing RARA transcriptional inhibition in the MCF7 breast cancer cell context differentially affects the growth response to high 'supraphysiological' RA. A-B. In the MCF7 breast cancer cell context RARA-target genes, such as CYP26A1 and RARB2, epigenetically repressed under 'physiological' RA conditions, can be reactivated by either high exogenous RA (as in the case of CYP26A1) (A), or a combination of high RA and chromatin drugs, such as 5-aza-2'-deoxycytidine (as in the case of RARB2) (B). RARB2 transcriptional reactivation is mediated by RARA, because it is counteracted by the RARA antagonist ER50891 (B). **C-D.** Stable expression of RARA403 in MCF7 cells ($MCF7^{403}$), which further exacerbates the inhibition of RARA transcriptional function (shown by reduced CYP26A1 induction by RA relative to $MCF7^{Ctrl}$) (C), makes cells grow significantly more in response to high exogenous RA (10^{-6}) (D). (*$p<0.05$, **$p<0.01$, ***$p<0.001$). Significance calculated by Student's t-test.

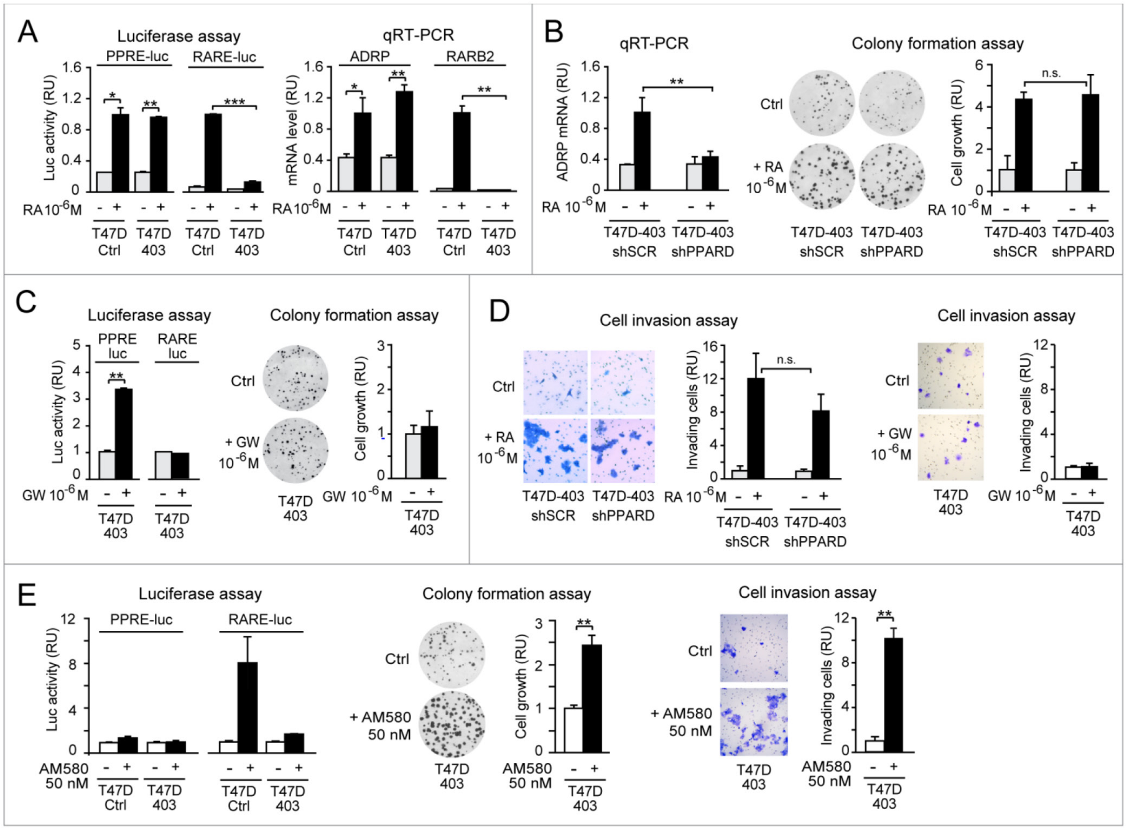

Supplementary Figure S4: PPARD is not involved in RA-induced T47D^{403} cell growth and invasion. A. High 'supraphysiological' RA (10^{-6}M) activates PPARD transcriptional function in both T47D^{Ctrl} and T47D^{403}, based on the induction of both PPAR-response element (PPRE)-driven luciferase (left) and ADRP, a prototypic PPARD-target gene (right). RA (10^{-6}M) induces RARE-luciferase (left) and RARB2 (right) only in T47D^{Ctrl} context. **B.** PPARD is not involved in RA-induced T47D^{403} cell growth, because stable PPARD knock down in T47D^{403} cells (T47D$^{403\text{-shPPARD}}$) does not counteract RA-induced growth (right), even if it hampers PPARD transcriptional function (lack of ADRP induction by RA) (left). **C.** The PPARD agonist GW501516 (GW), which activates PPARD, but not RARA (based on the induction of PPRE-luc, but not RARE-luc) (left), fails to promote T47D^{403} cell growth (right). **D.** PPARD is not involved in RA-induced T47D^{403} cell invasion, because PPARD knock down does not significantly counteract RA-induced cell invasion (left), and the PPARD agonist GW501516 does not promote it (right). **E.** The RARA agonist AM580, which activates RARA transcriptional function (assessed by RARE-luciferase) only in T47D^{Ctrl} (left), promotes both T47D^{403} growth (middle) and invasion (right), implicating a non-transcriptional RARA function in the RA cancer-promoting action. (ns=not significant, *$p<0.05$; **$p<0.01$; ***$p<0.001$). shSCR = shScrambled. Significance calculated by Student's t-test.

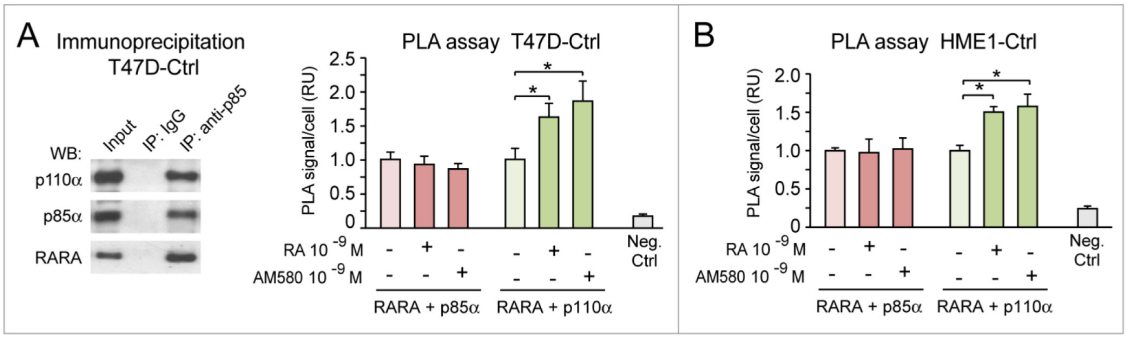

Supplementary Figure S5: Evidence that RA promotes RARA-PI3K interaction in both T47D and HME1 cell contexts. **A.** Immunoprecipitation with anti-p85α (PI3K regulatory subunit), followed by Western blot with anti-RARA, anti-p85α, or anti-p110α (PI3K catalytic subunit), shows the presence of a RARA/p85α/p110α complex in the T47D^{Ctrl} cell context in the presence of 'physiological' RA (left). Proximity ligation assay (PLA) confirms RARA binding to the PI3K subunits under 'physiological' RA conditions and shows that both 'supraphysiological' RA (10^{-9} M) and AM580 (10^{-9} M) significantly enhance the interaction between RARA and p110α (right). **B.** PLA assay shows that RA and AM580 promote RARA-p110α interaction also in the HME1 cell context. (*$p<0.05$). Significance calculated by Student's t-test.

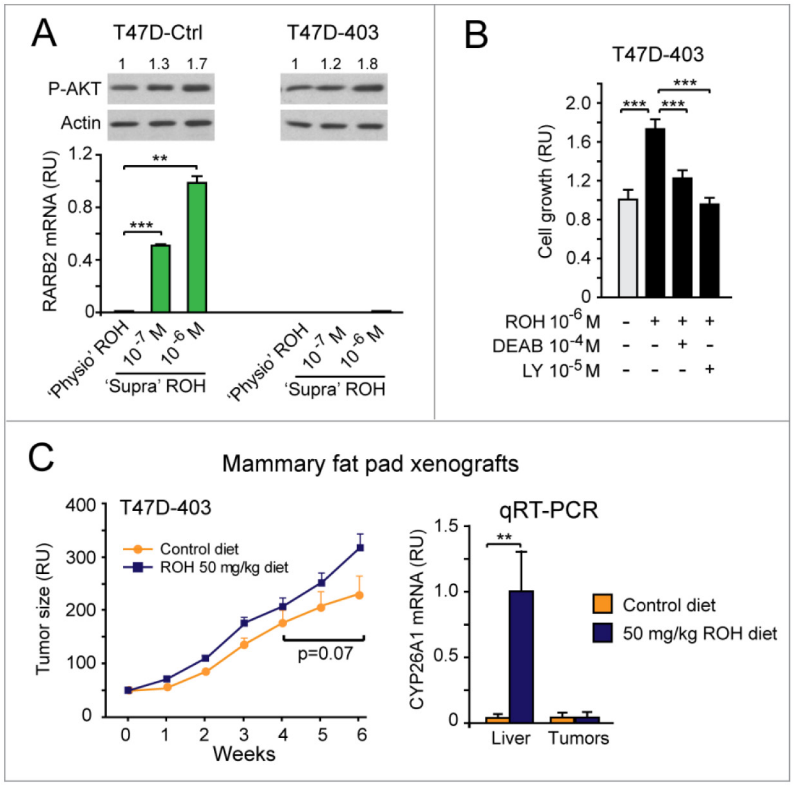

Supplementary Figure S6: Retinol promotes T47D^{403} cell growth by activating PI3K signaling. A. 'Supraphysiological' Retinol ('supra' ROH) promotes both RARA transcriptional signaling (assessed by induction of RARB2 transcript, bottom) and non-transcriptional signaling (assessed by induction of AKT phosphorylation, top) in T47D^{Ctrl}, but only RARA non-transcriptional signaling in T47D^{403}. **B.** T47D^{403} cell growth induced by 'supraphysiological' ROH (10^{-6}M) is counteracted by both the ALDH inhibitor DEAB and the PI3K inhibitor LY294002 (LY), thus indicating that ROH growth-promoting effects require both ALDH-mediated RA synthesis and PI3K activation (***p<0.001). **C.** A ROH-enriched diet promotes T47D^{403} xenograft tumor growth in nude mice (left) (p=0.07). The induction of CYP26A1 in liver tissue confirms ROH absorption from the diet, while lack of induction in T47D^{403} tumor tissue confirms RARA functional inhibition (right) (**p<0.01). Student's t-test was used for *in vitro* studies and ANOVA for *in vivo* studies.

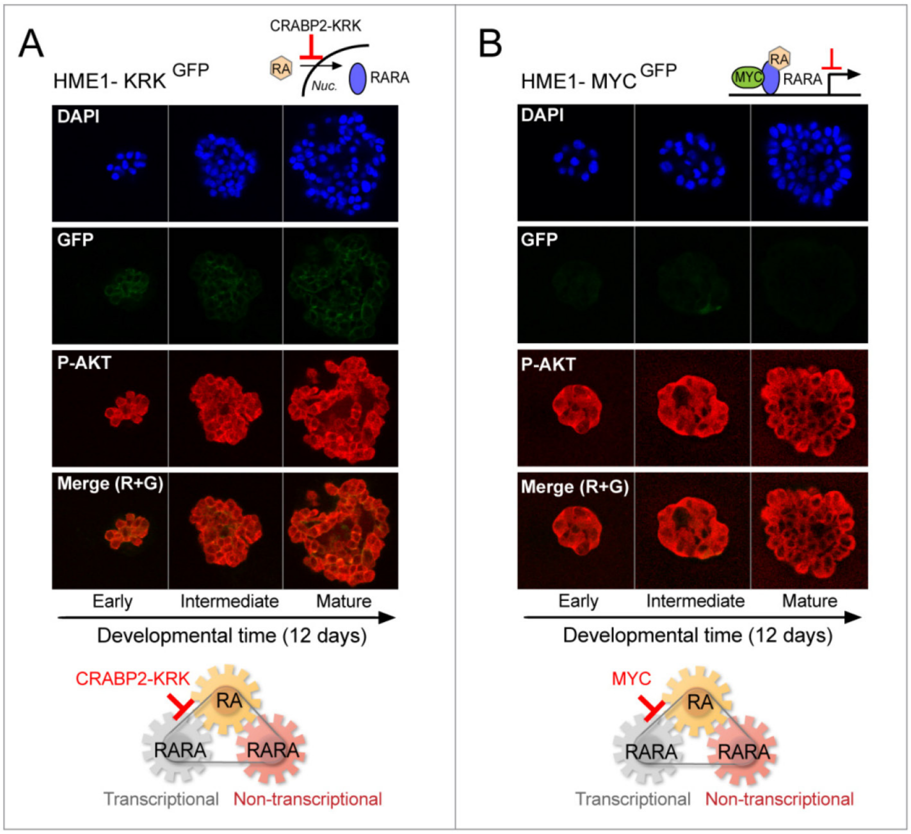

Supplementary Figure S7: Genetic alterations affecting RARA transcriptional function reveal physiological RA activation of PI3K/AKT signaling pathway during 3D mammary morphogenesis. A-B. 'Physiological' RA fails to induce transcriptional RARA signaling (no GFP), but keeps sustaining RARA-PI3K signaling (P-AKT) at all stages of 3D aberrant morphogenesis of both HME1$^{KRK\text{-}GFP}$, in which RA transport to the nucleus is affected by stable expression of the CRABP2-KRK mutant (A), and HME1$^{MYC\text{-}GFP}$ cells, overexpressing MYC, which can cooperate with RARA to repress RARE-containing genes (B).

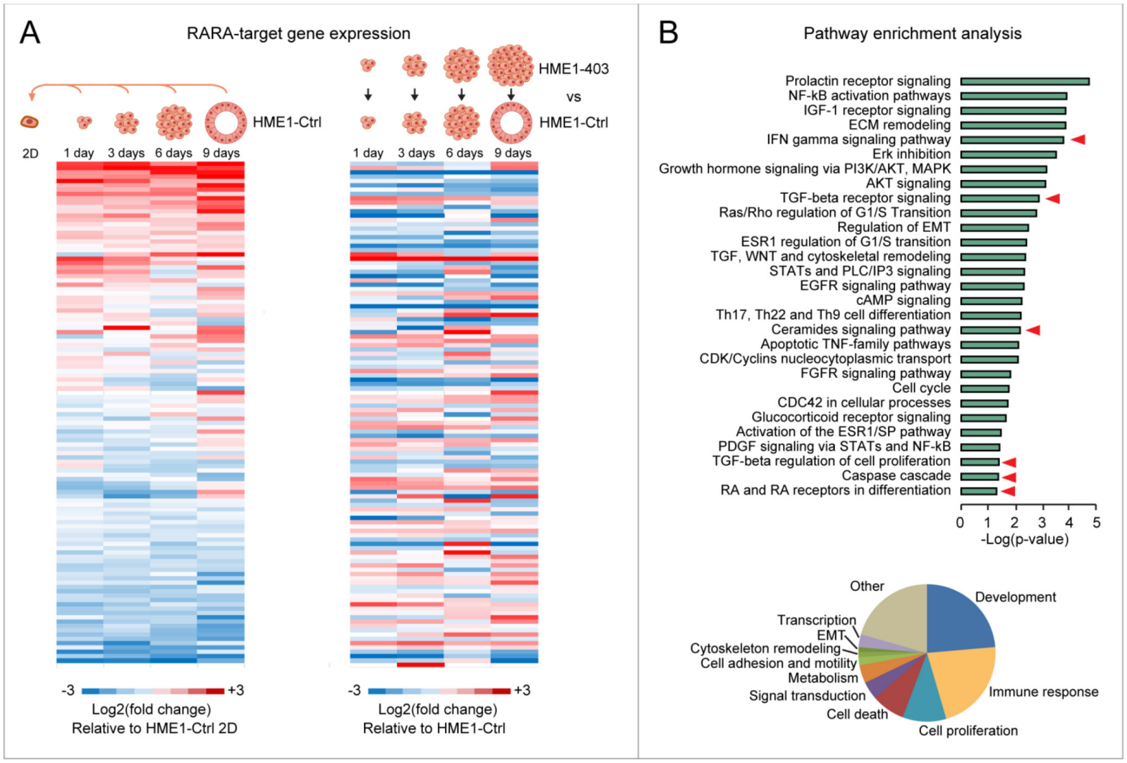

Supplementary Figure S8: Physiological RA, via RARA, dynamically modulates RARA-target gene expression during 3D mammary epithelial morphogenesis. A. The dynamic expression of the cohort of RARA-target genes in response to 'physiological' RA variation during HME1^{Ctrl} 3D morphogenesis (left) is affected during aberrant 3D HME1^{403} development (right). **B.** Metacore analysis shows that these RARA-targets are associated with signaling pathways (top) and cellular functions (bottom) relevant to mammary epithelial morphogenesis, including pathways known to be transcriptionally regulated by RARA (red arrows). Expression changes of RARA-target genes, which are part of larger gene clusters, contribute to the dynamics of signaling pathways shown in Figure 6.

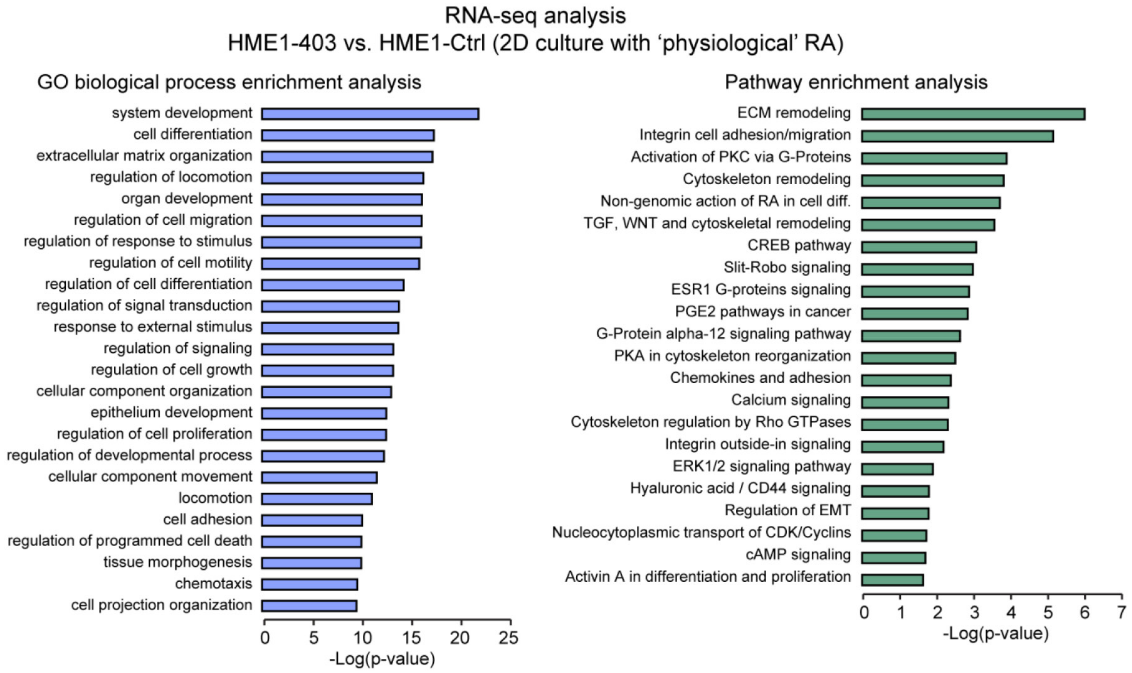

Supplementary Figure S9: HME1^{Ctrl} and HME1^{403} cells possess a 'built-in' potential to undergo normal or aberrant 3D morphogenesis. A. RNA-sequencing followed by Metacore enrichment analysis of the genes differentially expressed ($p<0.05$, absolute fold change >2) in HME1^{403} vs HME1^{Ctrl} cells grown in 2D culture shows deregulation of many Gene Ontology (GO) biological processes (left) and pathways (right), indicating that HME1^{Ctrl} and HME1^{403} cells possess a 'built-in' potential to undergo normal or aberrant 3D morphogenesis even before they are seeded in 3D culture.

Supplementary Table S1: RARA-target genes differentially regulated in T47D-403 vs. T47D-Ctrl in in the presence of 'physiological' or high 'supraphysiological' (10E-6 M) RA

See Supplementary File 1

Supplementary Table S2: RARA-target genes differentially modulated during 3D HME1-Ctrl and HME1-403 morphogenesis

See Supplementary File 2

Supplementary Table S3: Clusters of genes differentially modulated during 3D HME1-Ctrl and HME1-403 morphogenesis

See Supplementary File 3
